# Mapping the castor bean endosperm proteome revealed a metabolic interaction between plastid, mitochondria, and peroxisomes to optimize seedling growth

**DOI:** 10.3389/fpls.2023.1182105

**Published:** 2023-10-06

**Authors:** Thomas J. Wrobel, Dominik Brilhaus, Anja Stefanski, Kai Stühler, Andreas P. M. Weber, Nicole Linka

**Affiliations:** ^1^ Institute of Plant Biochemistry and Cluster of Excellence on Plant Sciences (CEPLAS), Heinrich Heine University, Düsseldorf, Germany; ^2^ Molecular Proteomics Laboratory, Biologisch-Medizinisches Forschungszentrum (BMFZ), Universitätsklinikum, Düsseldorf, Germany

**Keywords:** metabolism, storage reserve mobilization, inter-organellar interplay, metabolic network, proteomic study

## Abstract

In this work, we studied castor-oil plant *Ricinus communis* as a classical system for endosperm reserve breakdown. The seeds of castor beans consist of a centrally located embryo with the two thin cotyledons surrounded by the endosperm. The endosperm functions as major storage tissue and is packed with nutritional reserves, such as oil, proteins, and starch. Upon germination, mobilization of the storage reserves requires inter-organellar interplay of plastids, mitochondria, and peroxisomes to optimize growth for the developing seedling. To understand their metabolic interactions, we performed a large-scale organellar proteomic study on castor bean endosperm. Organelles from endosperm of etiolated seedlings were isolated and subjected to liquid chromatography-tandem mass spectrometry (LC-MS/MS). Computer-assisted deconvolution algorithms were applied to reliably assign the identified proteins to their correct subcellular localization and to determine the abundance of the different organelles in the heterogeneous protein samples. The data obtained were used to build a comprehensive metabolic model for plastids, mitochondria, and peroxisomes during storage reserve mobilization in castor bean endosperm.

## 1 Introduction

Castor bean (*Ricinus communis*) is a commercially important inedible oilseed crop in the Euphorbiaceae family, which was widely used as a model plant to study storage reserve mobilization (Beevers, 1961). Castor bean seeds contain on average about 64% storage oil, 18% storage proteins, and 1% starch in seed dry matter (Borek et al., 2015). Storage compounds are synthesized during seed filling phase of seed development. In case of castor bean, these reserves are accumulated exclusively in the endosperm surrounding the cotyledons of the embryo (Greenwood and Bewley, 1982). During germination, the stored oil, proteins, and starch are effectively metabolized in the endosperm. Their degradation products are exported from the endosperm tissue and absorbed by the embryo. Carbohydrates, amino acids, and nucleoside derivatives serve as essential nutrients to the developing cotyledons to promote growth and development until the seedling becomes photoautotrophic (Beevers, 1961; Kombrink and Beevers, 1983; Schobert and Komor, 1989). When the storage reserves are depleted, the endosperm detaches from the cotyledons and undergo developmentally regulated programmed cell death (Schmid et al., 1999; Greenwood et al., 2005).

Storage oil is deposited as triacylglycerol (TAG) in membrane-bound oil bodies in the castor bean endosperm. A massive conversion of the seed oil to carbohydrates during germination is a crucial process to support seedling establishment (Graham, 2008). This process was therefore intensively studied fifty years ago and became a classical textbook example of storage oil mobilization (Beevers, 1980). In particular, the discovery of peroxisomes involved in fatty acid degradation via β-oxidation and the glyoxylate cycle was a major achievement (Breidenbach and Beevers, 1967; Cooper and Beevers, 1969). The resulting oxoacids are converted to sucrose by gluconeogenesis involving peroxisomes and mitochondria (Kobr and Beevers, 1971).

The peculiarity of the castor bean oil is that it contains 90% of a rare hydroxylated fatty acid, namely ricinoleic acid (12-hydroxyoctadec-cis-9-enoic acid, C18:1-OH), which is extremely valuable for numerous industrial and chemical applications (Nitbani et al., 2022). Currently, castor bean seeds are the only commercial source of this unique fatty acids (Hutton and Stumpf, 1971). Over the past two decades, the molecular machinery involved in the production of hydroxy-fatty-acid containing TAG in castor bean has been identified to synthesize it in other oilseed crops (Nitbani et al., 2022). The catabolic pathway of this fatty acid, however, could not be conclusively elucidated, with the hydroxy group of ricinoleic acid forming a barrier to further degradation of ricinoleic acid by the core β-oxidation process (Hutton and Stumpf, 1971; Gerbling and Gerhardt, 1991).

Although most of the enzymatic steps involved in storage oil mobilization in castor bean endosperm have already been determined by the groups of Harry Beevers, Bernt Gerhardt, and Paul Karl Stumpf, the aim of our study was to elucidate the contribution of other metabolic reactions to this process, addressing the following questions: (1) Massive production of reducing equivalents are formed during the oxidation of fatty acids. How is peroxisomal redox homeostasis maintained in the endosperm tissue? (2) The seed-oil derived carbon skeletons are converted into sucrose, which is delivered exclusively to the embryo. Which other respiratory substrates are used as energy source by the endosperm tissue under this carbohydrate-limited condition? (3) The highly active metabolism of the endosperm is subject to a constant demand for cofactors, but also the maintenance of metabolic enzymes by protein synthesis and organelles by membrane expansion. Which reactions support these general cell functions in the endosperm?

To answer these questions, we focus on key organelles such as peroxisomes, mitochondria, and plastids because metabolic processes in eukaryotic cells are spatially distributed across different cellular compartments and thus interact with each other. We isolated these organelles from the endosperm tissue of etiolated seedlings and identified the organelle-specific proteome by using liquid chromatography-tandem mass spectrometry (LC-MS/MS).

Our large-scale organellar proteomic study allows us to assign the identified castor bean proteins to the previously described enzymatic activities. We are also able to elucidate the missing enzymatic steps in ricinoleic acid degradation and thus reconstruct a metabolic route for the complete degradation of ricinoleic acid in the endosperm tissue involving both peroxisomes and mitochondria. In addition, we describe here a comprehensive metabolic model for peroxisomes, mitochondria, and plastids, that explains how these organelles interact metabolically and how their organellar metabolism depends on each other during storage reserve mobilization in castor bean endosperm. These results provide a better understanding of the metabolic requirements for vigorous castor bean seed germination and seedling performance, which is crucial for crop improvement and breeding for higher-yielding castor bean cultivars.

## 2 Materials and methods

### 2.1 Plant growth conditions

Dry seeds of *Ricinus communis* var. *zanzibariensis* were surface-sterilized in a solution of 0.1% (w/v) 8-quinolinol in water for ten minutes and soaked in running tap water over night for seed imbibition. The imbibed seeds were placed on moist vermiculite and incubated at 30°C in the dark (Beevers and Breidenbach, 1974).

### 2.2 Fatty acid methylester (FAME) analysis

The ricinoleic acid content in the castor bean endosperm was determined using gas chromatography-mass spectrometry (GC-MS)-based fatty acid methyl ester (FAME) method (Hielscher et al., 2017). Endosperm tissue was dissected from dry seeds, 24h-imbibed seeds and 1- to 6-day old dark-grown seedlings by removing the embryo and roots with the blunt end of a scalpel blade, weighed and shock frozen in liquid nitrogen.

FAME analyses were generated by acid catalyzed methyl-ester formation using methanolic HCl as described by Hielscher et al. (2017). 20 mg endosperm was incubated at 90°C for one hour in 1 ml 3 N methanolic-HCl containing heptadecanoic acid (C17:0) as internal standard. The subsequent extraction of fatty acids from the sample was performed with 1 ml *n*-hexane and 1 ml 1% (w/v) sodium chloride. Samples were centrifuged at 2,000 *g* for five minutes. The resulting upper hexane phase was transferred into GC vials. The FAME extracts (1:100 diluted) were analyzed by GC Agilent 7890A gas chromatograph coupled to Waters GCT-TOF Premier mass spectrometer equipped with a Gerstel MPS2XL auto sampler.

Quantification and identification of the detected fatty acids was carried out with QuantLynx and MassLynx, respectively. Peaks were integrated and the resulting areas used for determination of relative changes in the abundance of the castor oil-specific ricinoleic acid. As the Ricinus endosperm started to gain fresh weight during seedling development due to water uptake, the fatty acid quantities were normalized to the fresh weight of an average endosperm. All samples were analyzed in three biological replicates.

### 2.3 Preparation of organelles from castor bean endosperm

The isolation of castor bean endosperm was performed according to Cooper and Beevers (1969) and Beevers and Breidenbach (1974). The protocols were modified by using the grinding buffer as described by Reumann et al. (2007). All steps were carried out on ice in a cold room (4°C) unless indicated otherwise. 30 g of endosperm tissue from 5-day old dark-grown Ricinus seedlings was harvested by removing the yellow cotyledons using the blunt side of a scalpel blade. The resulting endosperm was chopped using an onion chopper in 60 mL grinding buffer (170 mM Tricine pH X (KOH), 1 M Sucrose, 1% (w/v) BSA, 10 mM KCl, 1 mM MgCl_2_, 2 mM EDTA, 0.5% (w/v) PVP-40, and 5 mM DTT). The suspension was further homogenized using mortar and pestle. The homogenate was filtered through four layers cheesecloth. The crude extract was centrifuged at 1,200 *g* for 10 minutes to remove cell debris. The supernatant was carefully decanted into a new flask (approx. 40 mL). To separate the organelles, 6 mL of the obtained extract was loaded onto the top of a discontinuous sucrose gradient prepared in 20 mM Tricine-KOH (pH 7.5) and 1 mM EDTA. The density gradient consists of the following sucrose steps (from top to bottom): 5 ml 30% (w/w) sucrose, 3 ml 44% (w/w) sucrose, 5 ml 48% (w/w) sucrose, 5 ml 49% (w/w) sucrose, 1 ml 50% (w/w) sucrose, 2 ml 54% (w/w) sucrose, and 2 ml 60% (w/w) sucrose. The organelles were separated by ultracentrifugation at 105,026 *g* using a swing-out rotor for 3 hours. Four visible bands at the interface 30% − 44% (w/w) sucrose solution (fraction 1), 44% − 48% (w/w) sucrose solution (fraction 2), 48% − 49% (w/w) sucrose solution (fraction 3), and 50% − 54% (w/w) sucrose solution (fraction 4) were carefully collected, pooled, and stored at -80°C for further experiments.

### 2.4 Measurements of enzyme activity of organellar marker enzymes

The distribution of peroxisomes, mitochondria, and plastids within the four organellar fractions were examined using enzymatic marker proteins. All enzyme assays were performed photospectrometrically in a plate reader (SynergyH1, BioTek) at room temperature. For each sample three technical replicates were measured. Total protein concentration of the fractions was determined using the Pierce BCA protein assay kit (ThermoFisher Scientific). Enzyme activity was expressed as units per mg total protein. The activities of the following marker enzymes were analyzed: Catalase for peroxisomes (Breidenbach et al., 1968), fumarase for mitochondria (Nishimura and Beevers, 1981), and phosphoglycerate dehydrogenase for plastids (Benstein et al., 2013).

### 2.5 Isolation of organellar membranes

Membranes were isolated from the collected organellar fractions (F1-4) as described by Fujiki et al. (1982) and Reumann et al. (1995) with modifications. The obtained fractions were slowly diluted with hypo-osmotic buffer (20 mM HEPES-KOH, pH 6.8 and 0.8 mM MgCl_2_) and incubated on ice for 30 minutes to osmotically disrupt the organelles. The suspension was subjected to 10 freeze/thaw cycles (freezing in liquid nitrogen, thawing at room temperature) to lyse efficiently the organelles. After each thaw cycle the sample was homogenized by mixing. Membranes were sedimented by centrifugation at 100,000 *g* at 4°C for 1 h and washed in 100 mM sodium carbonate (pH 11.5) to remove membrane-associated proteins. A second centrifugation step (100,000 *g* at 4°C for 1 h) was performed to harvest the organellar membranes. The membrane pellet was resuspended in 20 mM HEPES-KOH, pH 6.8 and 0.8 mM MgCl_2_ and stored at -80°C for further experiments. Concentration of the membrane proteins were determined using Pierce BCA protein assay kit (ThermoFisher Scientific).

### 2.6 SDS-PAGE and immunoblotting

Membrane proteins from each organellar fraction (F1-4) were separated on an SDS-polyacrylamide gel and visualized using Colloidal Coomassie staining G-250 dye (Laemmli, 1970). To determine the purity of the organellar fractions via immunoblotting, membrane proteins separated by SDS-PAGE were transferred to a 0.2 µm polyvinylidene difluoride membrane, which was probed with primary antibodies against the following organellar membrane marker proteins: rabbit α-AOX (Agrisera, 1:1,000), rabbit α-BIP2 (Agrisera, 1:5,000), rabbit α-TIC40 (1:2,500), and rabbit α-PEX14 (Agrisera, 1:12,500). A horseradish peroxidase-conjugated goat α-rabbit IgG secondary antibody (Merck, 1:2,500) was used for chemiluminescence detection. The membrane was serially probed with antibodies to the indicated proteins. Imaging was performed with ImageQuant LAS 4000 Mini Bimolecular Imager (GE Healthcare) using exposure times between 1/8 and 15 seconds.

### 2.7 Proteome acquisition via mass spectrometry analysis

Total proteins and the enriched membrane proteins of the organellar fractions (F1-4) isolated from Ricinus endosperm tissue were analyzed by mass spectrometry (MS). Therefore, protein samples were loaded on an SDS-polyacrylamide gel, concentrated in the stacking gel, silver stained according to MS-compatible protocol, reduced, alkylated, and digested with trypsin. Peptides were extracted from the gel with 0.1% trifluoroacetic acid and subjected to liquid chromatography. For peptide separation an Ultimate 3000 Rapid Separation liquid chromatography system (Dionex; ThermoFisher Scientific) equipped with an Acclaim PepMap 100 C18 column (75 μm inner diameter x 50 cm length x 2 mm particle size from ThermoFisher Scientific) was used with a 140-minute LC-gradient. Mass spectrometry was carried out on an Obitrap Elite high-resolution instrument (ThermoFisher Scientific) operated in positive mode and equipped with a Nano electrospray ionization source. Capillary temperature was set to 275°C and source voltage to 1.5 kV. Survey scans were conducted in the orbitrap analyzer at a mass to charge (m/z) ranging from 350-1700 and a resolution of 60,000 (at 400 m/z). The target value for the automatic gain control was 1,000,000 and the maximum fill time 200 ms. The 20 most intense doubly and triply charged peptide ions (minimal signal intensity 500) were isolated, transferred to the linear ion trap (LTQ) part of the instrument and fragmented using collision induced dissociation (CID). Peptide fragments were analyzed using a maximal fill time of 200 ms and automatic gain control target value of 100,000. The available mass range was 200- 2000 m/z at a resolution of 5400 (at 400 m/z). Two fragment spectra were summed up and already fragmented ions excluded from fragmentation for 45 seconds.

### 2.8 Computational MS data analysis

For peptide and protein identification the acquired MS spectra were analyzed using the *MaxQuant* version 1.3.0.5 (MPI for Biochemistry, Planegg, Germany) with default parameters (Cox and Mann, 2008). Quantification was performed using the unlabeled quantification option of *MaxQuant*. The identified spectra were matched against the Ricinus proteome using the peptide search engine *Andromeda* (Cox et al., 2011). Only proteins containing at least two unique peptides and a minimum of three valid values in at least one group were quantified. A full list of all identified peptides from the proteome experiment is presented in **Supplemental Table S1**.

All identified Ricinus proteins were analyzed by bidirectional BLAST against the Arabidopsis proteome (Altschul et al., 1990). Organelle distribution within the collected fractions was assayed using a set of marker proteins. Proteins were assigned as organelle markers if the experimental localization of their *Arabidopsis* homologues in the SUBA 5.0 database (Hooper et al., 2017; Hooper et al., 2022) corresponded with their sequence-based localization prediction in Ricinus. We predicted protein localization to peroxisomes, mitochondria, and plastids manually and using the publicly available tools PPero, PredPlantPTS1, and TargetP (Emanuelsson et al., 2000; Reumann et al., 2012; Wang et al., 2015).

### 2.9 Statistical analysis and proteome deconvolution

Unless stated otherwise all data analyses were performed in R using the statistical package e1071 for support vector regression (http://www.R-project.org), ggplot2 for visualization (Wickham, 2009; Wickham, 2016; https://ggplot2.tidyverse.org) and NFM for Nonnegative Matrix factorization (Gaujoux and Seoighe, 2010; http://cran.r-project.org). To assign MS-identified proteins to a certain organellar fraction, several deconvolution approaches have been applied. Unsupervised deconvolution was performed by Nonnegative matrix factorization (NMF). We determined the rank of factorization using the Brunet- and Lee-Seung-algorithms for ranks from 2 to 10 with 50 repetitions (Gaujoux and Seoighe, 2010). Both algorithms were further constrained to sum to one in a sample, so that its value can be interpreted as the relative proportion of a fraction in a sample. We used random as well as double singular value decomposition for seeding (Boutsidis and Gallopoulos, 2008). The final factorization was performed 500 times with random seeding and a rank of 5. We determined the fractions specific for an organelle by scaling the coefficients per protein and clustered them via k-means with Euclidean distance. An enrichment of organelle-specific proteins in a cluster was determined via Fisher’s Exact Test and proteins specific for an organelle were used for classification. Proteins were assigned either to peroxisomes, plastids, mitochondria, and other organelles based on the fraction with the highest coefficient.

For supervised deconvolution we used the distribution of marker proteins normalized to its maximum in all samples. Quadratic programming as well as Support vector regression were constrained to yield positive values. Nu-type SVR was performed with a linear kernel and a set of Nu values (0.25; 0.5; 0.75; 1.0). To test the quality of prediction, deconvolution was repeated 500 times splitting the dataset into a training set containing 70% of the dataset and a testing set using the remaining 30% of the data. Proteins were associated to the organelle with the highest coefficient and consensus classification was achieved by simple majority vote of all four algorithms.

## 3 Results

### 3.1 Determining the seedling stage for storage oil mobilization

In castor bean (*R. communis)* the oil reserve is mainly stored in the endosperm surrounding the embryo. Upon germination, the endosperm mobilizes the storage oil to sucrose which is transported to the embryo to fuel post-germinative growth (Beevers, 1961). In particular, the peroxisomes of the endosperm are involved in the degradation of the seed-oil fatty acids via β-oxidation and the glyoxylate cycle (Breidenbach and Beevers, 1967; Cooper and Beevers, 1969). The core enzymes required for both metabolic pathways are well-characterized, but the complete catabolic pathway of ricinoleic acid (C18:1-OH) that comprises 85% of the castor seed oil remains enigmatic. Auxiliary enzymes are needed to metabolize this hydroxylated and unsaturated fatty acid in addition to the basic β-oxidation machinery. To identify the enzymes involved in the ricinoleic acid breakdown, we first determined the time point of the highest seed oil turnover rate in the endosperm tissue.

**Figure 1:**
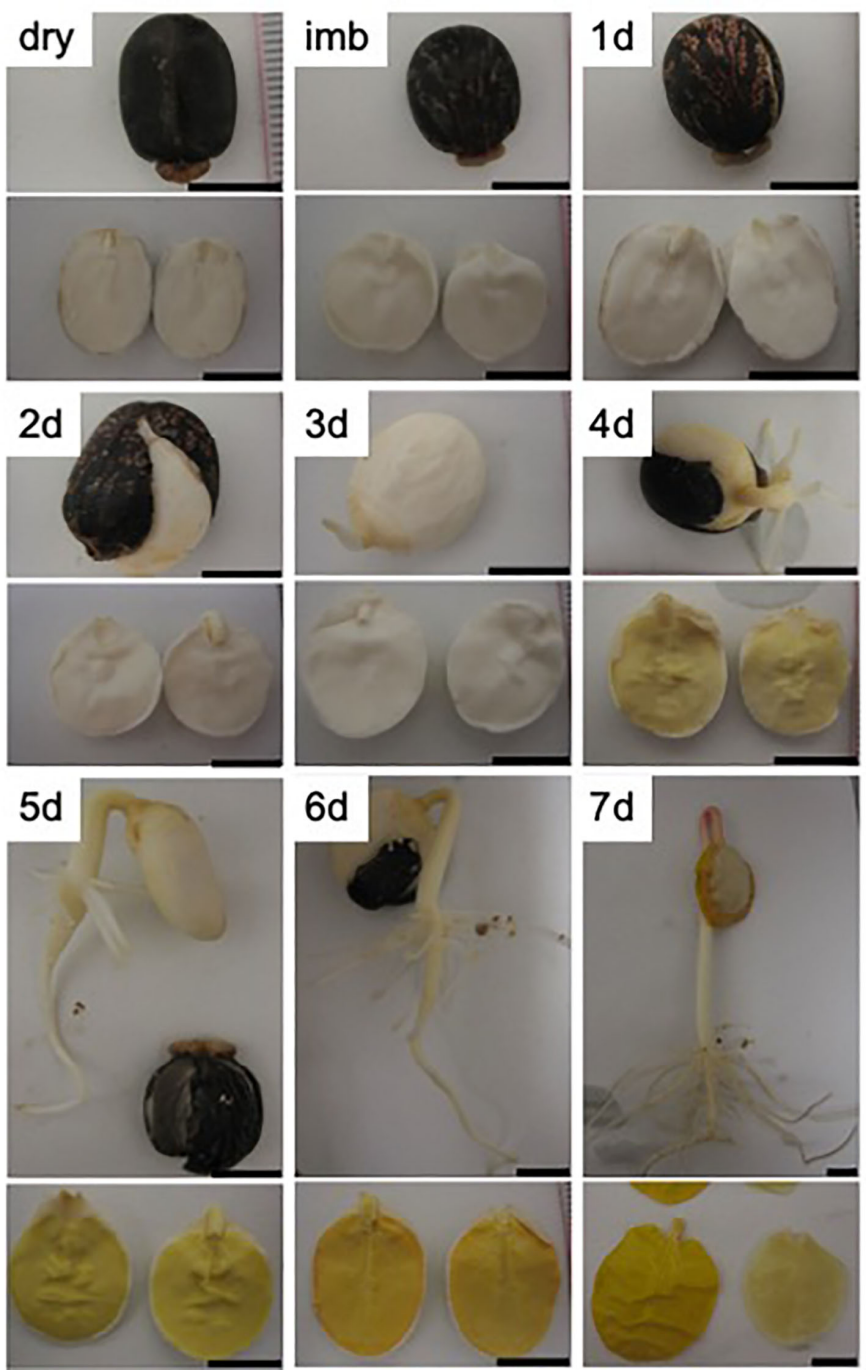
Endosperm morphology in seeds and germinating seedling of *R. communis*. Photographs were taken from the whole plant (*upper panel*) and two cotyledons embedded in the endosperm (*lower panel*) from mature seeds (dry), 24h-imbibed seeds (imb) and from 1- to 7-day old dark-grown castor bean seedlings (1-7d). Scale bar = 1 cm.

**Figure 2:**
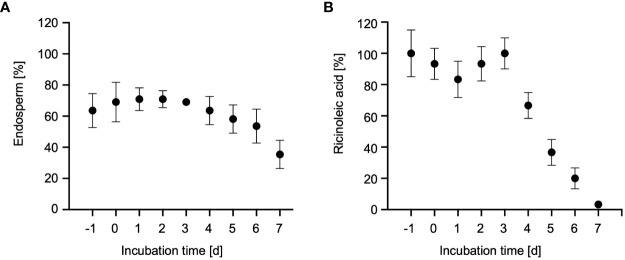
Storage oil mobilization in castor bean endosperm. **(A)** Relative fresh weight of the endosperm in dry seeds (-1d), imbibed seeds (0d) and 1- to 7-day old dark-grown castor bean seedlings (1-7d) as percentage of fresh weight of the whole plant. Data represent arithmetic means ± SD of 3 biological replicates. **(B)** Relative ricinoleic acid content of the endosperm from seeds and seedlings. Samples were normalized to the average fresh weight of on endosperm and expressed as percentages of their initial quantities determined in dry seeds (-1d). Error bars show standard deviations of the means of at least three biological replicates.

We harvested the endosperm from dry seeds, 24h-imbibed seeds and from 1- to 6-days old dark-grown castor bean seedlings (**Figure 1**). Recently imbibed and dry seeds with an intact seed coat had a bulky endosperm of waxy (oily) texture surrounding the cotyledons. One day after seed imbibition (DAI) the seed coat cracked. The radicula emerged at 2 DAI and the seed coat was cast off at 3 DAI. By 4 DAI the hypocotyl hook was visible, and the cotyledons turned yellowish. Within the next days, the hypocotyl elongated, the endosperm became increasingly slimy and detached from the cotyledons. The change in endosperm morphology from its initial waxy (oily) type to a soft slimy state can be followed by a decrease in the relative weight of the endosperm (**Figure 2A**), reflecting the depletion of storage oil in the endosperm tissue during seedling establishment.

To assess the onset of storage oil breakdown in the endosperm, we measured the content of ricinoleic acid (12-hydroxyoctadec-cis-9-enoic acid; C18:1-OH) using gas chromatography-mass spectrometry (GC-MS)-based fatty acid methyl ester (FAME) (Hielscher et al., 2017). Ricinoleic acid is the major fatty acid of seed oil stored in the castor bean endosperm where it constitutes up to 90% of the fatty acids found in triacylglycerols (Donaldson and Beevers, 1977). The level of this marker fatty acid started to decline after 3 DAI, until it was completely degraded at 7 DAI (**Figure 2B**). The highest decrease of ricinoleic acid in the endosperm occurred at 5 DAI (Muto and Beevers, 1974; Marriott and Northcote, 1975; Donaldson, 1977), reflecting the main phase of storage oil mobilization via peroxisomal fatty acid oxidation and the glyoxylate cycle. Thus, endosperm tissue of 5-day-old dark-grown castor bean seedlings was selected for subsequent organelle isolation.

### 3.2 Isolation of cell compartments from etiolated castor bean endosperm

**Figure 3:**
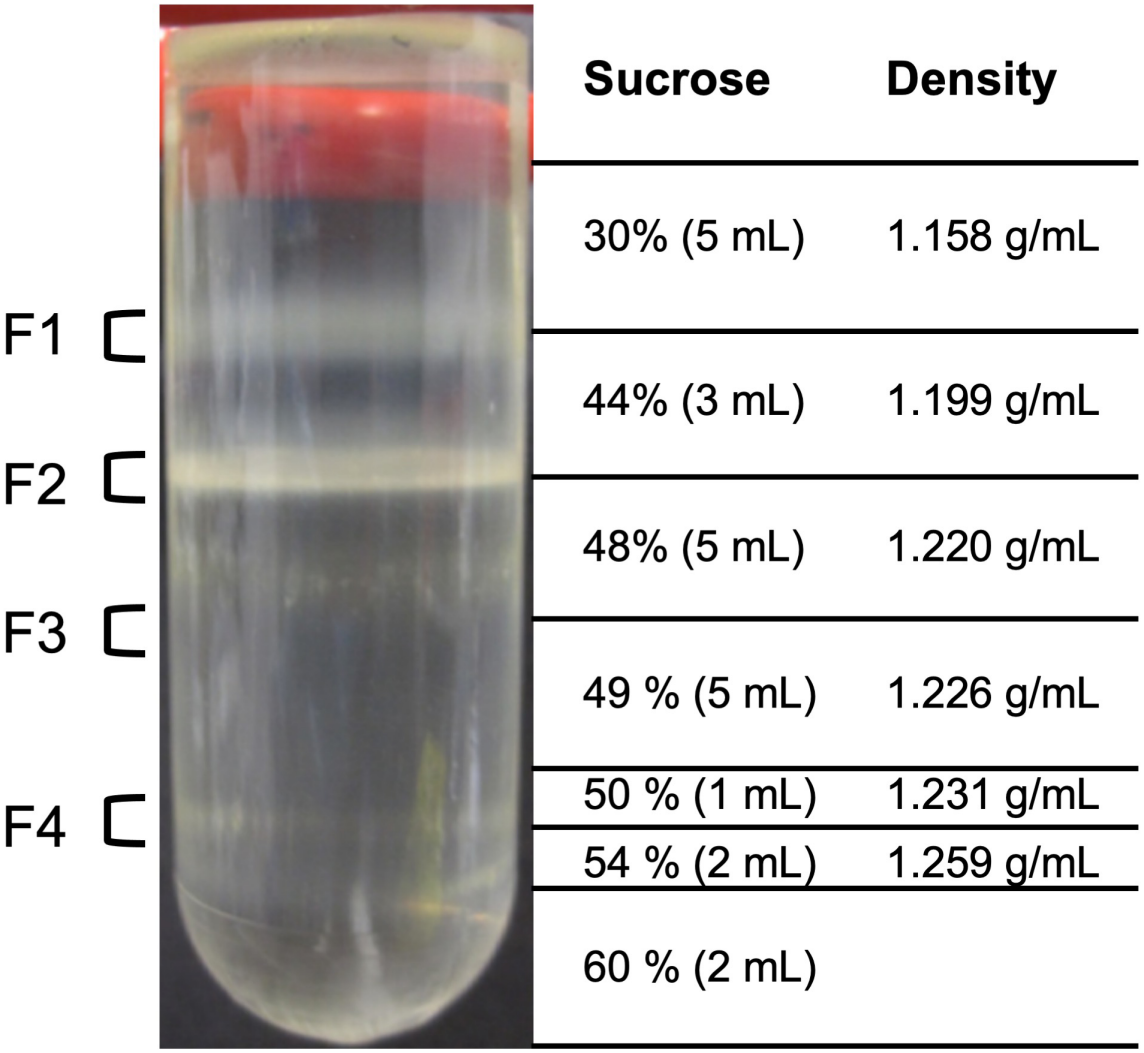
Isolation of organelles from etiolated castor bean seedlings. Four fractions of the sucrose density step gradient after centrifugation were taken from the gradient for various analyses at the interface 30% - 44% (w/w) sucrose solution (F1), 44% - 48% (w/w) sucrose solution (F2), 48% - 49% (w/w) sucrose solution (F3), and 50% - 54% (w/w) sucrose solution (F4).

Enzymes involved in endosperm reserve mobilization are distributed between several cell compartments, such as peroxisomes, mitochondria, and plastids. Therefore, we isolated organelles from endosperm of 5-day-old dark-grown castor bean seedlings using a stepwise (discontinuous) sucrose density gradient centrifugation method (Cooper and Beevers, 1969; Beevers and Breidenbach, 1974). The isolation of the cell compartments was performed in biological triplicates. After centrifugation, the endosperm crude extract was separated into four visible fractions (**Figure 3**), which were collected from the top to the base of the gradient: Fraction 1 (F1) at the interface 30% − 44% (w/w) sucrose, fraction 2 (F2) at 44% − 48% (w/w) sucrose, fraction 3 (F3) at 48% − 49% (w/w) sucrose, and fraction 4 (F4) at 50% − 54% (w/w) sucrose. The cell compartments of the castor bean endosperm should distribute with respect to their density within the following fractions: Peroxisomes have a high density of 1.25 g/mL and should sediment in F4, which represents the region between the density of 1.231 − 1.259 g/mL, plastids with a density of 1.19 g/mL in F3, mitochondria with a density of 1.23 g/mL in F2, and less dense organelles, such as Endoplasmic Reticulum (ER) and Golgi, in F1 (Breidenbach et al., 1968).

To test for the presence of peroxisomes, mitochondria, and plastids in the four fractions, we determined the activity of an enzyme that is known to be localized exclusively in each target organelle. We selected the following marker enzymes to provide information on the biochemical purity of the fractionated organelles: catalase for peroxisomes, fumarase for mitochondria, and phosphoglycerate dehydrogenase for plastids. The observed activities of marker enzymes (**Table 1**) point to an enrichment of the desired organelle is in the respective fractions. Although relatively low catalase activities (2-15%) were found in all fractions, the highest turnover was measured in peroxisomal fraction F4. Fumarase activities could be determined almost only in mitochondrial fraction F2, whereas the activities in F1 and F3 can be neglected (0.1-0.2%). Activities for plastidic marker enzyme were detected in fractions F2-4, but 6-fold higher turnover rates in plastidic fraction F3.

**Table 1:** Distribution of marker enzyme activities between the fractions.

	F1	F2	F3	F4
Enzyme	Enzyme activity (µmol/min mg protein in each fraction			
Catalase	9.1 ± 1.3	17.5 ± 2.4	72.6 ± 8.3	487.8 ± 36.4
Fumarase	0.2 ± 0.01	35.1 ± 0.08	0.1 ± 0.01	n.d.
Phosphoglycerate DH	n.d.	7.1 ± 1.4	109.7 ± 6.6	17.3 ± 1.8

Catalase served as peroxisomal marker, fumarase as the marker for mitochondria, and phosphoglycerate dehydrogenase represents plastids.

### 3.3 Enrichment of membrane proteins

**Figure 4:**
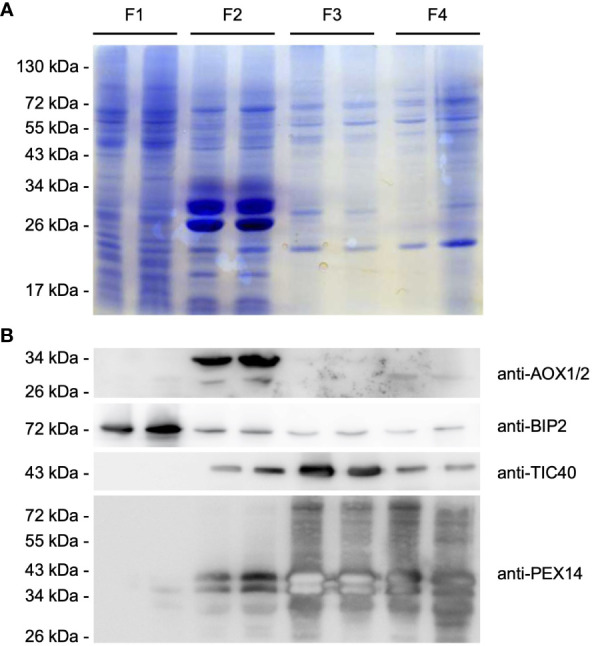
Purification of intracellular membranes from castor bean endosperm. Distribution of organellar membrane proteins within the organellar fractions (F1-F4) obtained after sucrose density gradient centrifugation. **(A)** SDS-PAGE analysis. Each lane was loaded with enriched membrane proteins from each pellet fraction after organelle disruption and ultracentrifugation (F1/F2: 25 µg, F3/F4: 15 µg). Equal loading of the protein gel within the fractions was monitored by staining with colloidal Coomassie G-250 dye. **(B)** Immunoblot analysis using antibodies raised against the following organelle membrane proteins: chloroplast TIC40, mitochondrial AOX, ER BIP2, and peroxisomal PEX14 proteins. Expected molecular weight for the corresponding castor bean proteins: 50.1 kDa for RcTIC40 (A3QSJ7), 39-41 kDa for RcAOX1/2 (B9RXE2/B9SO38), 73.3 kDa for RcBIP2 (B9RYP6), and 58.3 kDa for RcPEX14 (B9RB25). The membrane was serially probed with antibodies to the indicated marker proteins. The positions of molecular mass markers (in kDa) are indicated at the left.

Since many enzymes involved in the breakdown of endosperm reserve are bound to or embedded in the intracellular membranes, such as lipases, acyl-CoA synthetases or solute transport proteins, the four endosperm-derived organellar fractions were subjected to osmotic shock in combination with repeated freeze-thaw cycles to disrupt the organelles. Membranes from each organellar fraction (F1 to F4) were sedimented by centrifugation and washed with sodium carbonate to remove soluble proteins adhering to the membranes. Immunoassay was then conducted to evaluate the distribution of subcellular membrane proteins in the collected fractions (**Figure 4**).

Therefore, we used antibodies raised against the peroxisomal biogenesis factor 14 (PEX14), which resides in the peroxisomal membrane. The PEX14 antibody detected two present protein bands of ~50 and 55 kDa in F3 and F4, but also PEX14 signals were observed in F2. The recognized proteins were smaller than the expected molecular weight of 58 kDa for the castor bean PEX14. It is common for membrane proteins to behave anomalously during SDS-PAGE, because hydrophobic proteins do not bind SDS to the same extent as other soluble proteins, resulting in an abnormal charge-mass ratio (Rath and Deber, 2013). The presence of plastids was investigated using the antibody for the translocase of the 40 kDa translocase of the inner envelope membrane of plastids (TIC40). This antibody recognized the plastid-specific membrane protein with an estimated molecular weight of 50.1 kDa in F3, but also to an extent in F2 and F4. Alternative oxidases (AOXs) of the inner mitochondrial membrane as a mitochondrial marker were predominantly present in F2, indicating that mitochondria were exclusively assembled in this fraction. Antibody directed against the binding immunoglobulin protein 2 (BIP2) of the ER membrane detected a substantial amount of a 70 kDa protein in F1, corresponding to the calculated weight of the castor bean BIP. We observed low levels of the ER-specific protein in the high-density fractions.

Based on our immunoblot analysis, we were able to separate the cellular compartments based on their density as expected. Peroxisomes were enriched with high amounts in F4, plastids in F3, mitochondria in F2 and other compartments in F1, indicating good separation of each organelle during the isolation process. However, minor contamination with other cell compartments was observed in each organelle-enriched fraction.

### 3.4 Proteome analysis identification

We analyzed the proteome of the four density-gradient fractions (F1 to F4) as well as the corresponding membrane preparations (M1 to M4) to elucidate the metabolic pathways in the different organelles required in castor bean endosperm for optimal post-germinative growth. To this end, we subjected three independent biological samples of the total and membrane preparations to one-dimensional gel electrophoresis, followed by In-gel tryptic digestion and liquid chromatography-tandem mass spectrometry (LC-MS/MS). Proteome analysis of the total and membrane fractions in the four fractions (F1 to F4) revealed 2258 proteins that were present at least twice in the three replicates (**Supplemental Table S1**).

**Figure 5:**
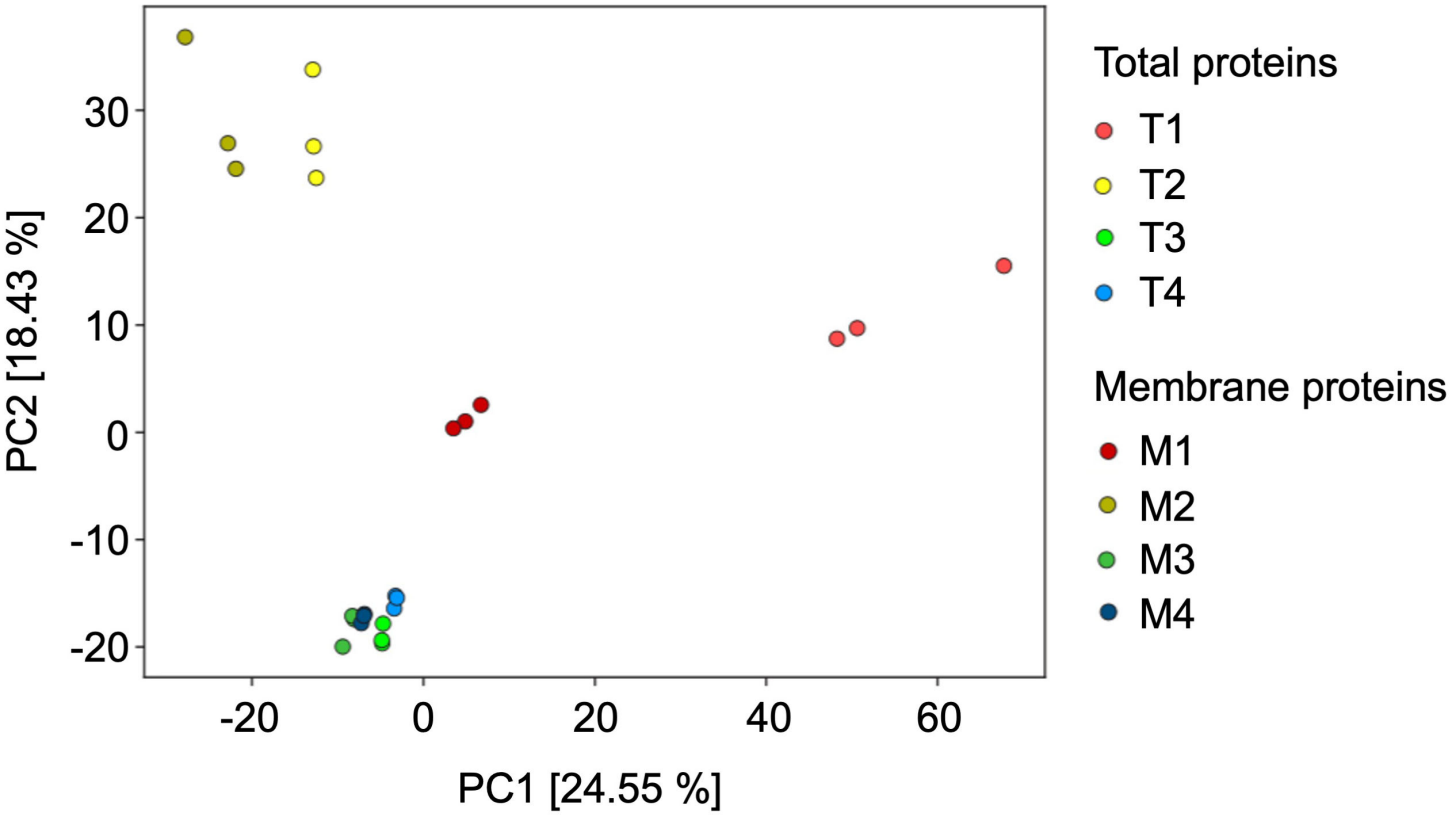
Principal Component Analysis of the Ricinus proteome of the fractions obtained from the sucrose density centrifugation, representing the total proteins (T1 to T4) and the enriched membrane proteins (M1 to M4). In this PCA plot each point represents the identified proteome of one biological experiment.

Principal component analysis (PCA) was used to compare the protein composition of all total and membrane fractions (**Figure 5**). In the PCA plot, the proteome of the peroxisome-enriched fraction F4 clusters closely together with the proteome of F3, pointing to very similar protein inventory between the two fractions in terms to total and membrane preparations. In contrast, the identified proteins in F1 and F2 are clearly separated and not co-clustered with the proteomes of F3 and F4, indicating a distinct protein composition with alterations between membrane and total samples. The outcome of the PCA analysis is consistent with the results from the marker enzyme activity and (**Table 1**) immunoblot analysis (**Figure 4**).

**Figure 6:**
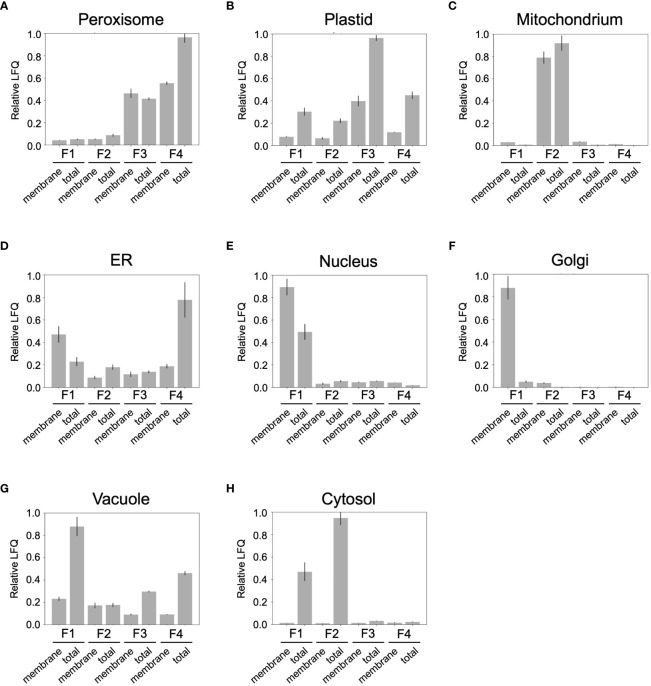
Distribution profile of compartment-specific marker proteins in the density-gradient fractions containing the total proteins (T1 to T4) and membrane proteins (M1 to M4). **(A)** peroxisomes; **(B)** plastid; **(C)** mitochondria; **(D)** ER; **(E)** nucleus; **(F)** Golgi; **(G)** vacuole; **(H)** cytosol. Relative LFQ represents the sum of Label Free Quantification (LFQ) values for all proteins belonging to an organelle relative to its maximal value.

To gain a deeper insight into the organelle distribution within the obtained fractions (F1 to F4), the abundance of organelle-specific Ricinus proteins was examined (**Figure 6**). To this end, we submitted the identified proteins of the castor bean endosperm to bidirectional BLAST against the Arabidopsis proteome. In the next step, we identified potential subcellular marker proteins from the 2258 Ricinus proteins. For this purpose, we first selected candidates whose Arabidopsis homologues have a unique subcellular location as defined by SUBA 5.0 via GFP-based experimental evidence GFP and/or proteomic approaches (Hooper et al., 2017; Hooper et al., 2022). Then, we used publicly available prediction tools PPero, PredPlantPTS1, and TargetP for peroxisomal, mitochondrial, and plastidic targeting sequences (Emanuelsson et al., 2000; Reumann et al., 2012; Wang et al., 2015) to define the set of Ricinus marker proteins. Ricinus proteins with contradicting classifications by the *in-silico* predictions were removed from the marker protein list. Our approach has resulted in a list of 160 organellar Ricinus proteins specifically located in peroxisomes (38 proteins), plastids (27 proteins), mitochondria (39 proteins), cytosol (17 proteins), vacuole (12 proteins), ER (11 proteins), Golgi (7 proteins), and nucleus (9 proteins) (**Supplemental Table S2**). Proteins associated with peroxisomes showed a distinct distribution in both F3 and F4. The highest abundance of peroxisomal proteins was found in the F4 sample (**Figure 6A**). Plastidic proteins were identified in all fractions (**Figure 6B**), although they were clearly enriched in F3. Mitochondrial proteins were specifically found in F2 (**Figure 6C**). ER-specific proteins were present in all fractions except membrane proteins, which were most abundant in F4 (**Figure 6D**). Nuclear and Golgi marker proteins were exclusively detected in F1 (**Figures 6E, F**). Vacuolar proteins were uniformly distributed in all fractions, with a strong peak for total proteins in F1 (**Figure 6G**). Cytosolic marker proteins were predominantly detected in F1 and F2 (**Figure 6H**). The distribution profile of organelle-specific marker proteins confirms our result of the marker enzyme activities (**Table 1**) and immunoblot analysis (**Figure 4**).

Overall, the density gradient centrifugation achieved a good separation of endosperm organelles, with an enrichment of mitochondria in F2, plastids in F3, and peroxisomes in F4. The other subcellular compartments such as ER, nucleus, Golgi, vacuole, and cytosol were abundant in F1.

### 3.5 Deconvolution of subcellular localization

The challenge was to reliably assign the MS-identified proteins to their correct subcellular localization due to a partial contamination of the organellar fractions by other cellular compartments. To overcome this problem, we applied deconvolution methods to assign our identified proteins to a specific subcellular localization within the endosperm tissue, even though the initial biochemical separation of organelles was not fully accomplished.

Deconvolution is an established method for the transcriptome-based dissection of cell proportions. Its use has been extended to the classification of cells from single cell transcriptomics (Abbas et al., 2009; Shao and Höfer, 2017; Chen et al., 2018; Duren et al., 2018). It assumes a linear correlation between the abundance of a cell and the measured gene expression in a sample which can be written as a matrix multiplication. We transferred this idea that protein abundance within a sample depends on the corresponding organelle concentration and applied computational deconvolution algorithms to determine the abundance of different organelles in heterogeneous samples.

We used linear regression (LR), quadratic programming (QP) and support vector regression (SVR) as supervised learning programs using marker proteins uniquely associated to peroxisomes, plastids, mitochondria, cytosol, vacuole, ER, Golgi, and the nucleus (**Supplemental Table S2**). Because these methods require prior knowledge of organelle proteins and their classifications is highly dependent on the number and quality of available marker proteins, we additionally analyzed our dataset using non-negative matrix factorization (NMF) as a means of unsupervised deconvolution. For the NMF analysis, the following categories were selected for classification: peroxisomes, mitochondria, plastids, and other (in which all other organelles were grouped).

Using the four deconvolution algorithms, we were able to group all 2258 identified endosperm proteins to a specific cell compartment. A defined subcellular localization was allocated when a protein was assigned to that organelle by at least three of the four deconvolution approaches (SVR, QP, LR, and NMF). In this way, we identified 141 peroxisomal proteins, 433 mitochondrial proteins, 222 plastidic proteins, 339 cytosolic proteins, 280 ER proteins, 268 Golgi proteins, 181 vacuolar proteins, and 123 nuclear proteins (**Supplemental Table S3**). 271 endosperm proteins could not be conclusive assign to a subcellular localization. This comprehensive protein inventory now allows us to draw the metabolic network between peroxisomes, mitochondria, and plastids in the castor bean endosperm during mobilization of storage reserve mobilization.

### 3.6 Metabolic pathways in etiolated castor bean endosperm

The castor bean endosperm plays an important role in supporting embryonic growth by supplying nutrients. To understand the metabolic role of these three organelles in this process and the extent to which they work together, we first examined our subcellular proteome of etiolated castor bean endosperm to determine which metabolic pathways take place in each compartment. Using the gene ontology term by UniProt database (The UniProt Consortium, 2023), we manually annotated the identified proteins and classified them into different biological processes (**Supplemental Table S4**). In addition to general functions, such as organelle biogenesis, protein import, protein quality import, and gene expression, peroxisomes, mitochondria, and plastids isolated from castor bean endosperm display primarily carbohydrate, lipid and amino acid metabolism, and other metabolic activities (**Supplemental Figure S1**). It should be noted, however, that the biological function of some of the identified proteins is unknown and therefore have not been assigned to a functional category. Nevertheless, these uncharacterized proteins may have a role in metabolism or other important functions in endosperm tissue. For each organelle, we also found some proteins with unclear localization that had not previously been described as peroxisomal, plastid, or mitochondrial proteins based on their function. Whether these proteins can possibly occur in multiple compartments remains to be clarified experimentally in the future.

Peroxisomal metabolism is thought to be very actively involved in the mobilization of storage oil at this stage of endosperm development (Beevers, 1961). And indeed, the identified core proteome represents the major peroxisomal metabolic pathways that is largely conserved in peroxisomes from various heterotrophic tissues (Quan et al., 2013; Pan et al., 2018). Most peroxisomal proteins were involved in processes related to the mobilization of storage oil to sucrose, such as lipid hydrolysis and fatty acid oxidation and glyoxylate cycle, as well as associated functions, including redox homeostasis and removal of ROS. Several solute transport proteins have been discovered that link these metabolic steps to the metabolism of the cell. Interestingly, two of the four key enzymes of the photorespiratory C2 cycle (glycolate oxidase and the glutamate/glyoxylate aminotransferase) were detected in peroxisomes, suggesting a different metabolic role in this non-photosynthetic endosperm tissue (Cooper and Beevers, 1969; Schnarrenberger et al., 1971; Schmitz et al., 2020). Other peroxisomal proteins are involved the catabolism of nitrogen-rich compounds, like polyamine, pseudouridine, and urate. However, anabolic reactions for synthesis of cofactor molecules and secondary metabolism also occur in the peroxisomes of castor bean endosperm, such as biosynthesis of auxin, jasmonic acid, phylloquinone, ubiquinone, and isoprenoids *via* the mevalonate pathway.

The mitochondrial proteome of castor bean endosperm contains a considerable number of proteins that are part of well-known mitochondrial functions, such as pyruvate oxidation, tricarboxylic acid (TCA) cycle, electron transport chain, and ATP synthesis (Lee et al., 2011; Salvato et al., 2014; Grabsztunowicz et al., 2020). The endosperm mitochondria also play a role in amino acid turnover, especially branched-chain amino acids, which act as respiratory substrates (Araújo et al., 2010; Binder, 2010; Kochevenko et al., 2012; Hildebrandt et al., 2015). In addition, numerous proteins are involved in lipid metabolism, such as mitochondrial fatty acid biosynthesis, synthesis of mitochondria-specific lipids (*e.g.*, cardiolipin), and lipid trafficking with the ER. In addition, proteins of the endosperm mitochondria are involved in the formation of various cofactors, like iron sulfur cluster, biotin, S-adenosylmethionine, ubiquinone, and folate. The presence of glycine decarboxylase and serine hydroxymethyltransferase in endosperm mitochondria, together with enzymes of the folate synthesis pathway, suggests a role for these key photorespiratory enzymes in one-carbon metabolism in addition to photorespiration and serine synthesis (Cossins and Sinha, 1967; Hanson and Roje, 2001; Schmitz et al., 2020). We have identified many different carrier proteins in the membranes of endosperm mitochondria that may be involved in metabolite exchange and link these metabolic pathways to those of the rest of the cell.

Since peroxisomes and mitochondria are predominantly involved in catabolism of storage oil and certain amino acids to produce sucrose and energy, endosperm plastids are active cellular factories (Baginsky et al., 2004; von Zychlinski et al., 2005; Grabsztunowicz et al., 2020). According to our proteome, the biosynthetic processes occur mainly in the plastids of the etiolated castor bean endosperm. The plastid proteome reported here covers a wide variety of enzymes that are involved in the *de novo* synthesis of fatty acids, lipids, amino acids, purine, pyrimidine, cofactors, and secondary metabolites (Neuhaus and Emes, 2000). For such an anabolic metabolism, the plastids require carbon skeleton precursors, ATP, and NADPH. In our proteome of endosperm plastids, we found enzymes of starch degradation, glycolysis, and the oxidative pentose phosphate pathway that provide the energy and reducing power necessary for these biosynthetic pathways. The identified carrier proteins of endosperm plastids might play a role in the shuttling of energy-rich molecules and metabolic intermediates between the plastid and cytosol. A comparison of the proteomes of other heterotrophic plastid types (Baginsky et al., 2004; von Zychlinski et al., 2005; Bräutigam and Weber, 2009; Grabsztunowicz et al., 2020) shows that the endosperm plastids exert a typical heterotrophic metabolism. An exception is the unexpected findings of components of the plastid ATP synthase complex and CO_2_-fixing ribulose-1,5-bisphosphate carboxylase/oxygenase (RubisCO) in our plastid proteome, but no subunits of the photosynthetic electron transport chain could be detected.

## 4 Discussion

The castor bean endosperm functions as an important storage tissue and provides the developing seedling with the required nutrients to become photoautotrophic. Mobilization of storage reserves requires the metabolic coordination of plastids, mitochondria, and peroxisomes to optimize post-germinative growth (Beevers, 1961). To this end, we conducted a large-scale organellar proteomic study of castor bean endosperm to understand the metabolic interactions of these cellular compartments and provide new insights into the metabolic network to fuel growth by degradation of the major fatty acid ricinoleic acid.

### 4.1 Metabolic role of peroxisomes in castor bean endosperm

**Figure 7:**
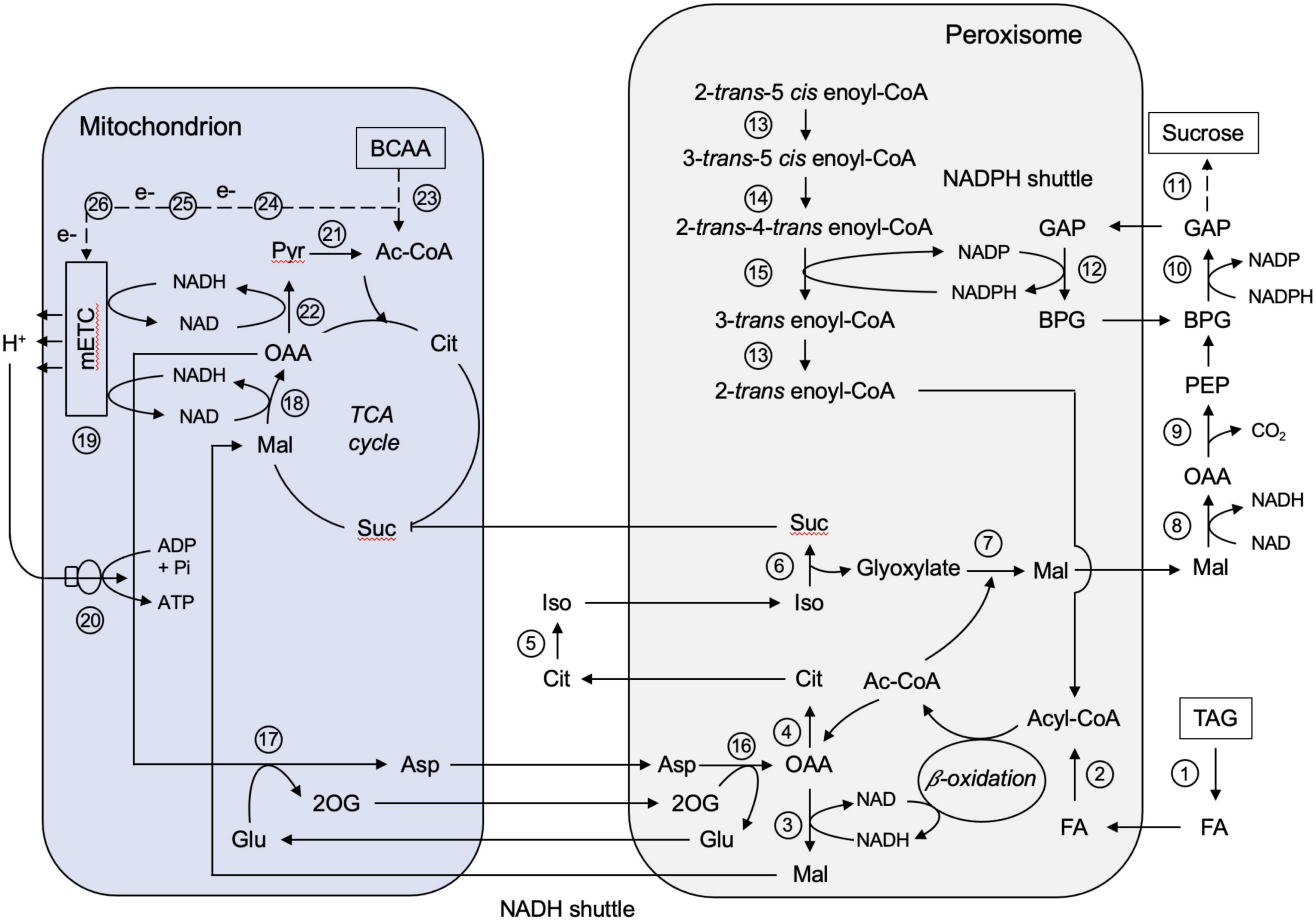
Metabolic interaction between peroxisomes and mitochondria during storage oil mobilization in germinating castor bean endosperm. Ac-CoA, Acetyl-CoA; Asp, aspartate; BCAA, branched-chain amino acids; BPG, 1,3-Bisphosphoglycerate; Cit, citrate; FA, fatty acid; GAP, Glycerinaldehyde-3-phosphate; Glu, glutamate; Iso, isocitrate; Mal, malate; OAA, oxaloacetate; 2OG, 2-oxoglutarate; PEP, phosphoenolpyruvate; Pyr, pyruvate; Suc, succinate; TAG, triacylglycerol. Enzyme numbers: 1: Lipase; 2: Long-chain acyl-CoA synthetase; 3: Peroxisomal malate dehydrogenase; 4: Citrate synthase; 5: Aconitase; 6: Isocitrate lyase; 7: Malate synthase; 8: Cytosolic malate dehydrogenase; 9: Phosphoenolpyruvate carboxykinase; 10: Cytosolic NAD-dependent glycerinaldehyde-3-phosphate dehydrogenase; 11: Enzymes involved in gluconeogenesis and sucrose biosynthesis; 12: Peroxisomal non-phosphorylating NADP-dependent glycerinaldehyde-3-phosphate; 13: Δ^3^,Δ^2^-Enoyl-CoA isomerase; 14: Δ^3,5^,Δ^2,4^-Dienoyl-CoA isomerase; 15: NADPH-dependent 2,4-Dienoyl-CoA reductase; 16: Peroxisomal aspartate aminotransferase; 17: Mitochondrial aspartate aminotransferase; 18: Mitochondrial malate dehydrogenase; 19: Mitochondrial electron transport chain; 20: Mitochondrial ATP synthase; 21: Mitochondrial pyruvate dehydrogenase; 22: Mitochondrial NAD-dependent malic enzyme; 23: Enzymes involved in BCAA degradation; 24: Isovaleryl-CoA dehydrogenase; 25: Electron transfer flavoprotein; 26: ETF:ubiquinone oxidoreductase.

One major metabolic process of castor bean endosperm is conversion of storage oil in form of TAG to sucrose, which requires an interplay with peroxisomes, mitochondria, and the cytosol (**Figure 7**). In the endosperm tissue, TAG mobilization starts with lipolysis (Muto and Beevers, 1974). We found three peroxisomal lipases involved in the release of fatty acids from glycerol backbone (**Figure 7**, No. 1). The known Patatin-like lipase (B9SVR2), an Arabidopsis homolog of Sugar-dependent protein 1 (SDP1; Eastmond, 2006), cooperates with a class 3 family lipase (B9T490). Both lipases cleave fatty acids at each of the sn-1 and sn-3 positions of TAG. The monoacylglycerol lipase (B9T6U9) hydrolyzes the third fatty acid. The fatty acids are then transported into the peroxisomes by the peroxisomal fatty acid transporter (PXA, B9RQ07). A castor bean homolog (LACS, A1X860) of the Arabidopsis long-chain acyl-CoA synthetase (Fulda et al., 2002) has been identified to activate the fatty acids to acyl esters (**Figure 7**, No. 2). The ATP required for this reaction is imported by the two peroxisomal membrane proteins (B9RF53, B9RTX9) that exhibit high sequence similarity to the peroxisomal adenine carrier in Arabidopsis (Arai et al., 2008; Linka et al., 2008). The core β-oxidation enzymes for the degradation of these acyl-CoAs were identified in the peroxisomes of the castor bean endosperm (**Figure 7**, No. 3), including (1) acyl-CoA oxidases (AXC, B9SH72, B9SGN6, B9T1J4, B9T4G5) oxidizing acyl-CoA to enoyl-CoA, (2) multifunctional protein (MFP, B9RT76, B9RKN5) catalyzing the hydration and oxidation of enoyl-CoA to β-keto-acyl-CoA, and (3) β-Keto-acyl-CoA thiolase (KAT, B9S554, B9RWL7) cleaving β-keto-acyl-CoA into acetyl-CoA and a two carbons shorter acyl-CoA, which can re-enter the next β-oxidation cycle. In case of unsaturated fatty acids, a set of auxiliary enzymes are required to provide the β-oxidation machinery with the proper substrates, such as the Δ^3^,Δ^2^-Enoyl-CoA isomerase (ECI, B9SR30, B9SR76; **Figure 7**, No. 13), Δ^3,5^,Δ^2,4^-Dienoyl-CoA isomerase (DCI, B9RM93; **Figure 7**, No. 14), and NADPH-dependent 2,4-Dienoyl-CoA reductase (DECR, B9RU96, B9RBG0; **Figure 7**, No. 15).

The oxidation of fatty acid leads to high levels of hydrogen peroxide (H_2_O_2_). We found the two major H_2_O_2_ scavengers in endosperm peroxisomes that protects the organelle and thus the rest of the cell from oxidative damage. Catalase (CAT, B9R8N4, B9S6U0) catalyzes the conversion of H_2_O_2_ to water and oxygen with a high rate (Beevers and Breidenbach, 1974; Theimer and Theimer, 1975). In addition, peroxisomes contain an ascorbate peroxidase (APX, B9SXV4) and a monodehydroascorbate reductase (MDAR, B9S635). APX oxidizes H_2_O_2_ to water and oxygen and reduces ascorbate to monodehydroascorbate, which is then recycled to ascorbate by MDHAR using NADH as a cofactor. Both activities have been reported in the membrane of castor bean peroxisomes (Bowditch and Donaldson, 1990; Bunkelmann and Trelease, 1996; Karyotou and Donaldson, 2005). Because APX has a higher affinity for H_2_O_2_ than catalase (Klapheck et al., 1990), this antioxidative system guarantees effective H_2_O_2_ detoxification and thus prevents deleterious H_2_O_2_ leakage from peroxisomes.

The major end products of the peroxisomal β-oxidation is acetyl-CoA, which is further converted by the peroxisomal glyoxylate cycle to malate (Beevers, 1961; Breidenbach and Beevers, 1967; Cooper and Beevers, 1969; Gerhardt and Beevers, 1970). We detected the entire set of glyoxylate cycle enzymes in the endosperm tissue, which are orchestrated between peroxisomes, cytosol, and mitochondria: peroxisomal malate synthase (MLS, B9RAK0; **Figure 7**, No. 7), peroxisomal citrate synthase (CSY, B9T369; **Figure 7**, No. 4), peroxisomal isocitrate lyase (ICL, B9SUS2; **Figure 7**, No. 6) and cytosolic aconitase (ACO, B9SXB6; **Figure 7**, No. 5). Succinate produced by the glyoxylate cycle can enter the mitochondrial TCA cycle by the mitochondrial dicarboxylate/tricarboxylate carrier (DTC, B9T1E7, Picault et al., 2002; Toleco et al., 2020). Malate generated from glyoxylate can be directed into gluconeogenesis to build the hexose units for sucrose biosynthesis. In the cytosol, malate is converted to phosphoenolpyruvate (PEP) via oxaloacetate by the NAD-dependent malate dehydrogenase (cMDH, B9T5E3; **Figure 7**, No. 8) and the phosphoenolpyruvate carboxykinase (PCK, B9R6Q4, B9SSD5; **Figure 7**, No. 9). The key enzymes for sucrose biosynthesis, like sucrose-phosphate synthase (SPS, B9T123) and sucrose phosphate phosphatase (SPP, B9SDM9), were also present in the cytosolic fraction of our endosperm proteome (**Figure 7**, No. 11). The flux of small mono-/dicarboxylic acids into and out of peroxisomes thought to occur via a non-selective channel termed peroxisomal membrane protein of 22 kDa (PMP22, Charton et al., 2019). However, no orthologue could be found in the peroxisomal proteome of endosperm tissue.

The activity of NAD-dependent dehydrogenase during the fatty acid breakdown leads to the production of NADH, which must be re-oxidized to NAD to sustain high turnover rates of β-oxidation. We found a peroxisomal NAD carrier (PXN, B9R8X3, B9SLI5) that might be involved in preloading this organelle with NAD (Agrimi et al., 2012; Bernhardt et al., 2012). This transport protein is not able to mediate an NAD/NADH exchange for NADH oxidation outside peroxisomes (van Roermund et al., 2016). A malate-aspartate shuttle was described as NAD regenerating system in castor bean endosperm that operates between peroxisomes and mitochondria (Cooper and Beevers, 1969; Mettler and Beevers, 1980). The simpler malate/OAA shuttle was not proposed, because isolated castor bean mitochondria cannot take up OAA (Chappell and Beevers, 1983). The enzymes required for the operation of this redox shuttle were identified in our proteomic approach, including peroxisomal malate dehydrogenase (pMDH, B9S7S1, B9T172; **Figure 7**, No. 3), mitochondrial malate dehydrogenase (mMDH, B9S977, B9SE47; **Figure 7**, No. 18), peroxisomal aspartate aminotransferase (pASP, B9RS47, B9RKN9; **Figure 7**, No. 16), and mitochondrial aspartate aminotransferase (mASP, B9SW33; **Figure 7**, No. 17). The transport proteins mediating the exchange of these metabolites across the inner mitochondrial membrane were detected in our proteome: The uncoupling protein carrier (UCP, B9SPF2) importing glutamate against aspartate (Monné et al., 2018) and the dicarboxylate/tricarboxylate carrier (DTC, B9T1E7) importing malate (or succinate) against 2-oxoglutarate (Picault et al., 2002; Toleco et al., 2020). The benefit of malate-aspartate redox shuttle is to link peroxisomal fatty acid degradation to mitochondrial ATP formation by the transfer of reducing equivalents between these organelles.

During peroxisomal β-oxidation NADPH is necessary for the removal of fatty acid double bonds by the 2,4-Dienoyl-CoA reductase (DECR, B9RU96, B9RBG0; **Figure 7**, No. 15), suggesting suitable redox shuttles for the NADPH regeneration (Donaldson, 1982). As known from yeast and mammals (Henke et al., 1998; van Roermund et al., 1998), a 2-oxoglutarate/isocitrate shuttle involving NADP-linked isocitrate dehydrogenases in peroxisomes (gICDH, B9SR98, NADP-dependent) and in the cytosol (cICDH, B9SMI9) is postulated. The presence of such a shuttle implies that peroxisomal isocitrate is not primarily used by the glyoxylate cycle to produce glyoxylate, but some of it is also converted by isocitrate dehydrogenase to 2-oxoglutarate for NADPH generation during β-oxidation (Corpas et al., 1999). Based on our endosperm proteome an alternative mechanism can be proposed that may operate in parallel with the NADP redox shuttle described above. We discovered a NADP-dependent non-phosphorylating glyceraldehyde-3-phosphate dehydrogenase (NP-GAPDH, B9SBX6; **Figure 7**, No. 12) in peroxisomes that could provide NADPH inside the peroxisomal matrix by oxidation of glycerinaldehyde-3-phosphate (GAP) generated during gluconeogenesis. Such a NADP shuttle has been described to export NADPH from the chloroplast to the cytosol in Arabidopsis (Rius et al., 2006). The advantage of this NADP redox shuttle is that it does not compete with the glyoxylate cycle enzyme isocitrate lyase for the peroxisomal isocitrate pool. The oxidative phase of the pentose phosphate pathway (OPPP), mediated by glucose-6-phosphate dehydrogenase (G6PDH) and 6-phosphogluconate dehydrogenase (6PGDH) may additionally be involved in NADPH provision for unsaturated fatty acid degradation. However, for this pathway we could only identify the NADP-dependent 6-phosphogluconate dehydrogenase (6PGDH, B9RVA7) in endosperm peroxisomes (Corpas et al., 1998; Meyer et al., 2011; Hölscher et al., 2014; Hölscher et al., 2016).

### 4.2 Deciphering a long-awaited pathway for ricinoleic acid degradation

**Figure 8:**
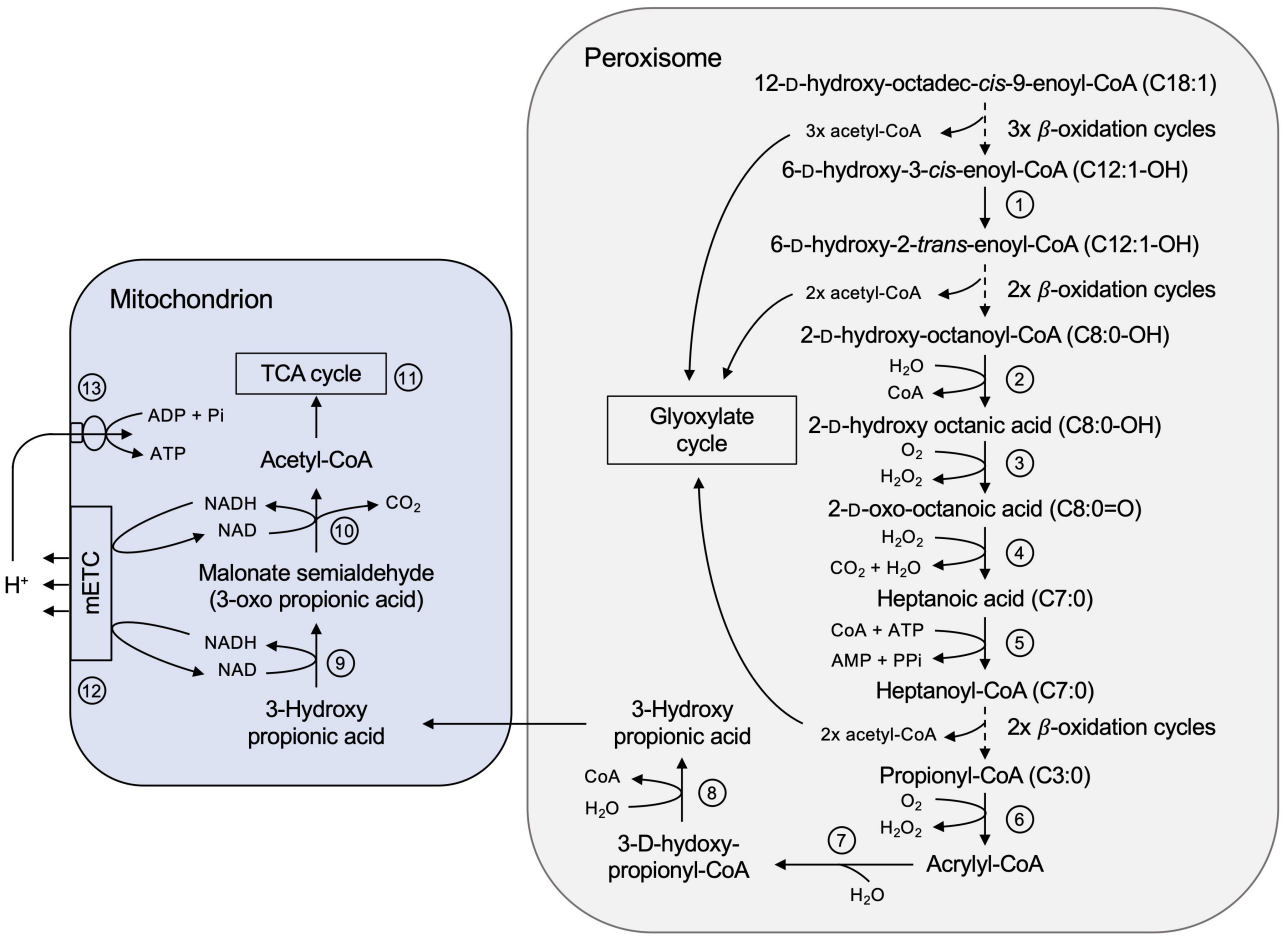
Complete degradation pathway of ricinoleic acid in the castor bean endosperm involving peroxisomes and mitochondria. In the peroxisomal lumen the hydroxylated fatty acid (C18:1-OH) is activated to 12-D-hydroxyoctadec-cis-9-enoyl-CoA to enter its degradation pathway. Enzyme numbers: 1: Δ^3^,Δ^2^-enoyl-CoA isomerase; 2: Acyl-CoA thioesterase; 3: α-2-Hydroxy acid oxidase; 4: enzyme-independent oxidative decarboxylation; 5: Short-chain acyl-activating enzyme; 6: Acyl-CoA oxidase; 7: Monofunctional enoyl-CoA hydratase; 8: 3-Hydroxy acyl-CoA hydrolase; 9: 3-Hydroxy acid dehydrogenase; 10: Malonate semialdehyde dehydrogenase; 11: Enzymes of the TCA cycle; 12: Mitochondrial electron transport chain; 13: Mitochondrial ATP synthase.

Using our castor bean endosperm proteome, we were able to elucidate the still enigmatic players in the catabolic pathway of the unusual hydroxylated fatty acid ricinoleic acid, which makes up to 80-90% of the castor oil (Hutton and Stumpf, 1971; Marriott and Northcote, 1975). This proposed model (**Figure 8**) is supported by intermediates of the ricinoleic acid degradation detected by HPLC analyses of acyl-CoA esters and free fatty acids on germinating castor bean seedlings (Hutton and Stumpf, 1971; Gerbling and Gerhardt, 1991).

The complete breakdown of ricinoleic acid to acetyl-CoA encounters two obstacles. After the first three rounds of β-oxidation, the intermediate 6-D-hydroxy-5-*cis*-enoyl-CoA (12:1-OH) is not recognized by the core enzyme activities due to a *cis*-double bond at an odd-numbered carbon. It must be converted to 2-*trans*-enoyl-CoA, a common substrate of β-oxidation. According to the peroxisomal proteome we suggest that the peroxisomal Δ^3^,Δ^2^-enoyl-CoA isomerase (ECI, B9SR30, B9SR76; **Figure 8**, No. 1) catalyzes the required *cis-to-trans* isomerization of the double bond. The multifunctional protein (MFP) of the core β-oxidation cycle might also contribute to isomerize the *cis*-configuration of the double bond, since a Δ^3^,Δ^2^-enoyl-CoA isomerase activity has been biochemically demonstrated for this enzyme (Gerhardt, 1993).

The second stop occurs after two more β-oxidation cycles leading to the formation of α-D-2-hydroxy-octanoyl-CoA (C8:0-OH). The hydroxyl group at the C-2 carbon position (α-carbon) prevents further degradation via the β-oxidation enzymes. When ricinoleic acid was offered as a substrate to cell extracts or enriched peroxisomes isolated from etiolated castor bean seedlings, 2-oxo-octanoic acid (8:0) appeared in the assay in a H_2_O_2_-producing manner (Hutton and Stumpf, 1971; Gerbling and Gerhardt, 1991). We identified in the endosperm peroxisomes an Acyl-CoA thioesterase (ACH, B9RDR2; **Figure 8**, No. 2), yielding free fatty acids by CoA cleavage, and two α-2-hydroxy acid oxidases (HAOX, B9ST69, B9ST75; **Figure 8**, No. 3), coupling the oxidation to 2-oxo-octanoic acid (8:0) with the formation of H_2_O_2_. In human, two peroxisomal HAOX isoforms oxidize medium- and long-chain α-2-hydroxy fatty acids that are present in sphingolipids metabolism during lipid turnover (Jones et al., 2000; Esser et al., 2014). Isolated peroxisomes provided with racemic α-2-hydroxy octanoic acid were able to metabolize the L-isomer, but also the D-isomer, concluding that the identified HAOX enzymes are not stereo-specific (Gerbling and Gerhardt, 1991).

The resulted 2-oxo-octanoic acid (8:0), however, cannot be fed into β-oxidation, because the hydroxyl group forms a barrier to further degradation. Biochemical studies demonstrated that this unesterified C8-intermediate was converted to heptanoyl-CoA (7:0) and CO_2_, suggesting oxidative decarboxylation as a mechanism (Gerbling and Gerhardt, 1988; Gerbling and Gerhardt, 1991). We could not identify a specific enzyme that might catalyze such a reaction and therefore assume an enzyme-independent decomposition of this 2-oxo fatty acid to heptanoic acid in the presence of H_2_O_2_ (**Figure 8**, No. 4). It was reported that various 2-oxo acids such as pyruvic acid and 4-methyl-2-oxopentanoic acid can be spontaneously decarboxylated by H_2_O_2_ (Bunton, 1949; Melzer and Schmidt, 1988; Vlessis et al., 1990; Lopalco et al., 2016; Onada et al., 2018; Hansen et al., 2021). This would explain why peroxisomes from different plant species, such as hypocotyl of etiolated mung bean seedlings, were also able to efficiently degrade ricinoleic acid without containing a specific enzyme repertoire (Gerbling and Gerhardt, 1988). To enter β-oxidation again, the resulted short-chain fatty acid is then activated by a short-chain acyl-activating enzyme (SACS, B9RE68; **Figure 8**, No. 5), which has been discovered in our peroxisomal proteome. The final product of this catabolic pathway are acetyl-CoA and the odd-numbered acyl-CoA ester propionyl-CoA.

We identified respective proteins in our castor bean endosperm proteome that could convert propionyl-CoA via acrylyl-CoA to 3-hydroxypropionate in a modified β-oxidation pathway in peroxisomes. This is consistent with the previous reports of acryloyl-CoA and 3-hydroxypropionate as intermediates of propionyl-CoA degradation pathway found in either whole plants or isolated peroxisomes (Hatch and Stumpf, 1962; Gerbling and Gerhardt, 1989). Propionyl-CoA is oxidized to acrylyl-CoA, which could be performed by β-oxidation acyl-CoA oxidases (**Figure 8**, No. 6). Subsequently, monofunctional enoyl-CoA hydratases (ECH, B9R8I2, B9RZR7; **Figure 8**, No. 7) catalyze the hydration of acryloyl-CoA to 3-hydroxypropionyl-CoA. The action of a 3-hydroxy acyl-CoA hydrolase (HADH, B9SKJ5; **Figure 8**, No. 8) would lead to the formation of 3-hydroxypropionate. The key enzymes for further breakdown are not found in peroxisomes, however, in mitochondria, suggesting an interplay of both organelles. 3-hydroxypropionate is an intermediate of the mitochondrial branched-chain amino acid degradation pathway, where it is metabolized to acetyl-CoA and CO_2_. Acetyl-CoA can enter the mitochondrial TCA cycle to be completely oxidized to CO_2_ and H_2_O in two consecutive reactions catalyzed by the 3-hydroxy acid dehydrogenase (HDH, B9S5X5; **Figure 8**, No. 9) and the malonate semialdehyde dehydrogenase (MMSDH, B9RX74; **Figure 8**, No. 10). With our proteome study, we can assign the corresponding enzymes to this catabolic pathway of propionyl-CoA. The transport of this intermediate from peroxisomes to mitochondria might be mediated by non-selective pore-forming channels, such as PMP22 and the closely related PMP22 protein Mpv17 (Wi et al., 2020), which has been detected in the mitochondrial endosperm proteome (B9RKQ0).

### 4.3 Metabolic role of mitochondria in castor bean endosperm

The metabolic role of mitochondria during storage lipid mobilization in the castor bean endosperm is (1) the conversion of succinate to malate via the TCA cycle for sucrose biosynthesis, (2) the regeneration of NAD for continuous action of peroxisomal fatty acid oxidation via the malate/aspartate shuttle (as described above), and (3) the generation of ATP by oxidative phosphorylation to supply the cellular ATP demand. Enzymes involved in the mitochondrial electron transport chain and the ATP synthesis were present in the endosperm mitochondria (**Supplemental Table S4**; **Figure 7**, No. 19-20).

Regarding the TCA cycle, only certain reactions of this pathway are required to form malate from succinate in mitochondria (Canvin and Beevers, 1961). Nevertheless, the enzymatic activities of the entire TCA cycle have been found in isolated endosperm mitochondria (Cooper and Beevers, 1969; Millhouse and Wiskich, 1986). Using our mitochondrial proteome, we were able to confirm this. All TCA enzymes, with the exception of mitochondrial citrate synthase (mCSY), were present: mitochondrial aconitase (mACO, B9T2U5), mitochondrial NAD-dependent isocitrate dehydrogenase (mIDH, B9S0K1), 2-oxoglutarate dehydrogenase complex (ODC, B9SVA1 and B9SR46), the succinyl-CoA synthetase (SCoAL, B9SV11), succinate dehydrogenase complex (SDH, B9R7F6, B9SWW3, and B9SLS5), fumarase (FUM, B9SAW4), and mitochondrial malate dehydrogenase (mMDH, B9S977 and B9SE47; **Figure 7**, No. 18). This indicates that the mitochondrial TCA cycle fulfills additional functions in endosperm mitochondria. A fundamental process of the mitochondrial TCA cycle is the oxidation of acetyl-CoA to carbon dioxide (CO_2_) which is associated with the generation of NADH and FADH_2_. which deliver their electrons to the electron transport chain for ATP synthesis (Sweetlove et al., 2010; Zhang and Fernie, 2018). The reoxidation of these reducing equivalents by the electron transport chain drives mitochondrial ATP synthesis (**Figure 7**, No. 19-20).

Acetyl-CoA, the substrate of the TCA cycle, can be provided by several enzymatic pathways. Based on our mitochondrial proteome we propose several routes allowing optimal supply of acetyl-CoA. First, pyruvate can be imported into mitochondria via the mitochondrial pyruvate carrier (MPC, B9RXD3, Le et al., 2021) and then metabolized to acetyl-CoA by mitochondrial pyruvate dehydrogenase complex (PDC, B9RT82, B9S2H9, B9RFW4, B9S5V2, B9SL87; **Figure 7**, No. 19). Second, we found in the endosperm mitochondrial alanine aminotransferases (mAlaAT, B9T1D1 and B9SZ94), which provide pyruvate by transamination reaction (Fuji and Yoshii, 1995). Third, a mitochondrial NAD-dependent malic enzyme (NAD-ME, B9RDN0; **Figure 7**, No. 22) of the endosperm proteome could generate pyruvate via the oxidative decarboxylation of malate (Tronconi et al., 2008).

While most of the reduced carbon from storage lipid degradation goes into sucrose biosynthesis, we assume that endosperm mitochondria prefer BCAAs as respiratory substrates for ATP production (Hildebrandt et al., 2015). Most of the enzymes involved in the complex BCAA degradation were found in endosperm mitochondria (**Figure 7**, No. 23). The initial catabolic steps of leucine, isoleucine, and valine are identical and begin with the transamination of the BCAAs by the mitochondrial BCAA transaminase (BCAA-T, B9R6U0, Angelovici et al., 2013). The resulting branched-chain 2-oxoacids are further metabolized to acyl-CoA molecules via oxidative decarboxylation, which is catalyzed by the branched-chain α-keto acid dehydrogenase complex (BCKDH, B9S2P3 and B9SBN1, Hildebrandt et al., 2015). Subsequently, isovaleryl-CoA dehydrogenase (IVDH, AT3G45300; **Figure 7**, No. 24) oxidizes methyl-branched acyl-CoAs, transferring the electrons into the mitochondrial electron transport chain at the level of ubiquinone via electron transfer flavoprotein (ETF, B9SA46 and B9STH5; **Figure 7**, No. 25) and ETF:ubiquinone oxidoreductase (ETF-QO, B9SU11; **Figure 7**, No. 26) (da Fonseca-Pereira et al., 2021). Leucine catabolism proceeds with a carboxylation reaction catalyzed by methylcrotonyl-CoA carboxylase (B9SFG9 and B9RYH1) to produce 3-methyl-glutaconyl-CoA (Alban et al., 1993; Anderson et al., 1998; Lucas et al., 2007). For the next steps, it is still controversial whether they are localized in plant mitochondria (Binder, 2010; Kochevenko et al., 2012). In case of castor bean endosperm, we suggest the further degradation requires the involvement of peroxisomal β-oxidation as well as other auxillary enzymes, such as monofunctional enoyl-CoA hydratase (ECH, B9R8I2 and B9RZR7), hydroxymethylglutaryl-CoA lyase (B9RU86), 3-hydroxyacyl-CoA dehydrogenase (B9SQH3), and 3-hydroxybutyryl-CoA dehydratase (HADH, B9SKJ5). The breakdown of isoleucine and valine leads to propionyl-CoA as end product, which is further converted to 3-hydroxypropionate inside peroxisomes and then finally to acetyl-CoA in mitochondria, as described above for the degradation of ricinoleic acid. Thus, the BCAAs not only serve as a source of electrons for the mitochondrial electron transport chain to generate ATP, but also provide acetyl-CoA to maintain the operation of the TCA cycle under C-limiting conditions.

### 4.4 Metabolic role of plastids in castor bean endosperm

The role of plastids in castor bean endosperm has been extensively studied with respect to fatty acid and lipid biosynthesis for seed filling (Reid et al., 1977; Vick and Beevers, 1978; Miernyk and Dennis, 1982). In contrast, the metabolic function of plastids during post-germinative germination is largely unknown. Based on our proteomic analysis, we argue that plastids are indirectly involved in the degradation of storage oil by synthesizing fatty acids, amino acids, nucleotides, and cofactors (**Supplemental Table S4**). These molecules support growth and development of the embryo, but also contribute to the maintenance of the endosperm functions.

**Figure 9:**
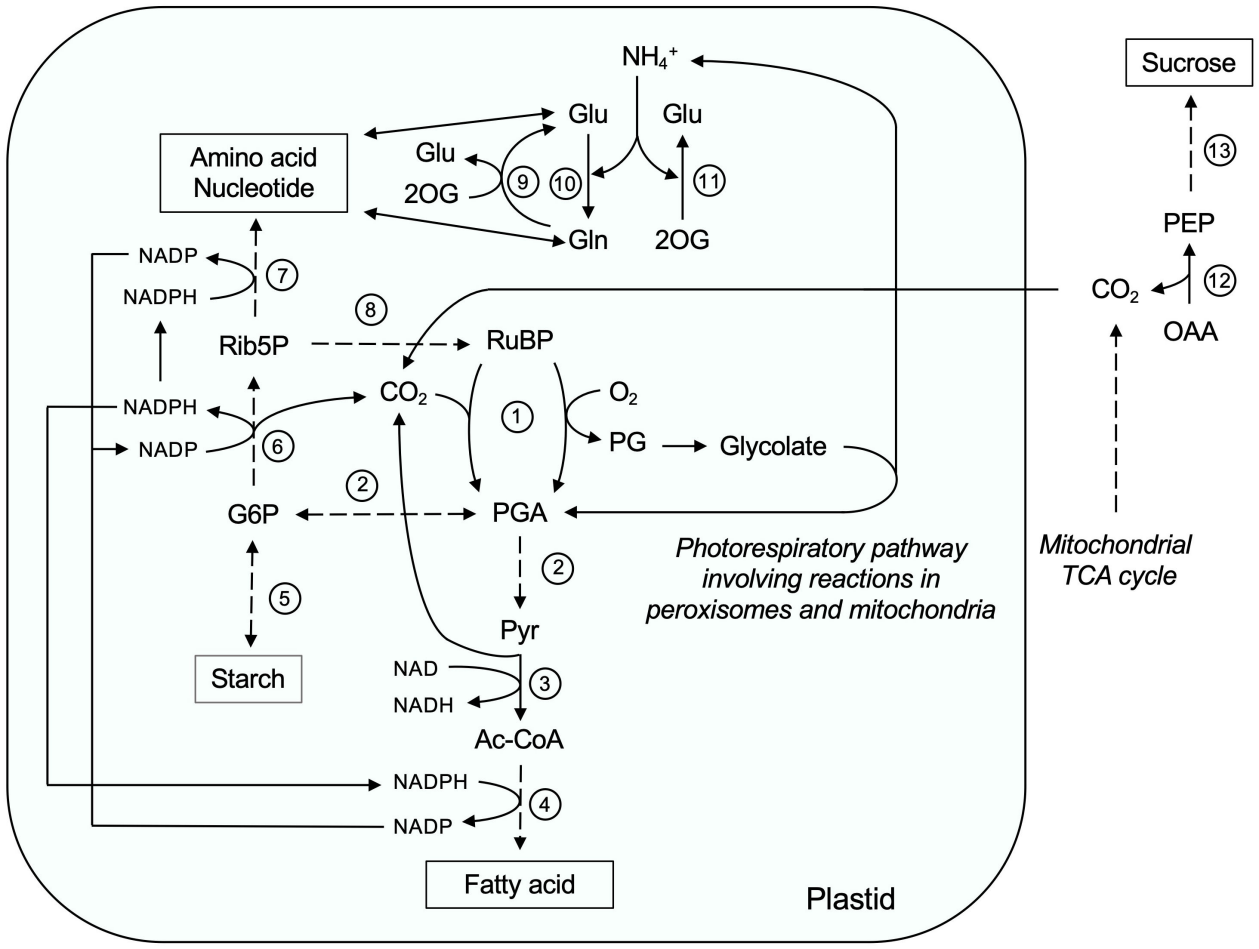
The role of plastids during storage oil mobilization in germinating castor bean endosperm. Ac-CoA, Acetyl-CoA; G6P, Glucose-6-phosphate; Gln, Glutamine; Glu, Glutamate; OAA, oxaloacetate; 2OG, 2-Oxoglutarate; PEP, phosphoenolpyruvate; PG, 2-Phosphoglycolate; PGA, 3-Phosphoglycerate; Pyr, Pyruvate; Rib5P, Ribose-5-phosphate; RuBP, Ribulose-1,5-bisphosphate. Enzyme numbers: 1: RubisCO; 2: Glycolytic enzymes; 3: Plastidic pyruvate dehydrogenase; 4: Plastidic fatty acid synthase complex; 5: Enzymes involved in starch synthesis and degradation; 6: Enzymes involved in the oxidative part of the pentose phosphate pathway; 7: Enzymes involved in amino acid and nucleotide biosynthesis; 8: Phosphoribulokinase; 9: Glutamate synthetase; 10: Glutamine synthetase; 11: NAD-dependent glutamate dehydrogenase; 12: Phosphoenolpyruvate carbxykinase; 13: Gluconeogenesis and sucrose biosynthesis pathway.

Endosperm plastids are still capable of synthesizing fatty acids and lipids during post-germinative growth (**Supplemental Table S4**; **Figure 9**, No. 4). Feeding experiments with labeled acetate as a precursor for fatty acids have been shown that germinating castor bean endosperm synthesizes membrane lipids *de novo* rather than using stored lipid components directly (Donaldson, 1976). This fact indicates that there is a continuous need in terms of membrane expansion and organelle biogenesis in the endosperm tissue upon germination. We detected the components of the acyl carrier protein (ACP)-dependent fatty acid synthase (FAS) complex in the plastid proteome. In this process, a two-carbon unit is added to the growing acyl chain at each cycle until a C16:0 molecule is produced. This fatty acid can be extended to C18:0 by the β-ketoacyl-ACP synthase synthase II (KAS2, A6N6J4) and/or desaturated to C18:1 by a stromal stearoyl-ACP desaturase (SAD, B9T0X0). The plastid-produced fatty acids serve as the acyl building blocks for the assembly of membrane lipids. We identified plastid proteins involved in the synthesis of plastid membrane lipids such as galactolipid, sulfolipid, and glycerolipid (**Supplemental Table S4**). However, some of these fatty acids are exported to the ER for membrane lipid synthesis, which is required for other cellular compartments, such as peroxisomes and mitochondria (Li-Beisson et al., 2013). However, plastidic membranes require an intensive supply of lipid precursor from the ER and a component of the lipid transfer complex (TGD2, AT3G20320) is present in castor bean plastid.

Acetyl-CoA is substrate of the plastid fatty acid synthase complex, which is generated from pyruvate through the action of the pyruvate dehydrogenase complex (PDH, B9ST02, B9RZN2, B9RNK3, and B9SLH2; **Figure 9**, No. 3). Our proteome supports the idea that plastids from germinating endosperm contain all enzymes of the glycolic pathway for the conversion of glucose-6-phosphate to pyruvate (**Supplemental Table S4**; **Figure 9**, No. 2), except for triosephosphate isomerase (B9STC9) and phosphoglycerate mutase (B9S5D4) annotated by our deconvolution methods as unclassified or nuclear proteins, respectively (**Supplemental Table S3**). The spatial separation of glycolysis and gluconeogenesis between plastid and cytosol allows both reciprocal metabolic pathways to occur simultaneously (Kobr and Beevers, 1971; Nishimura and Beevers, 1979). To provide glucose-6-phopshate, heterotrophic plastids are able to import glucose-6-phopshate from the cytosol. Since we could not detect a plastidic glucose-6-phopshate/phosphate translocator in our proteome study, and the cytosolic hexose-phosphate pool is mainly used for sucrose biosynthesis, the plastids themselves could generate glucose-6-phosphate by phosphorolytic degradation of starch via starch phosphorylase (PHS, B9SJB6; **Figure 9**, No. 5) and would thus be self-sufficient. Starch as storage carbohydrate is synthesized during endosperm development and starch granules are present in the plastids of mature castor bean seeds (Bianchini and Pacini, 1996). However, we have also found enzymes involved in starch biosynthesis in plastids of germinating endosperm, such as ADP-glucose pyrophosphorylase (AGP, ADP-glucose pyrophosphorylase) and starch branching enzyme (SBE, B9T792), indicating that both processes, starch turnover and synthesis, take place in endosperm plastids (Reibach and Benedict, 1982; Goldner and Beevers, 1989).

An alternative pathway to provide pyruvate for plastidial fatty acid biosynthesis could be via RubisCO. The activity of this enzyme has been demonstrated in the endosperm of germinating castor beans (Benedict, 1973; Osmond et al., 1975; Gillen et al., 1976) and the corresponding protein subunits were found in our plastid proteome (B9SYG1 and G1D766; **Figure 9**, No. 1). In this non-photosynthetic tissue, RubisCO can operate without Calvin-Benson-cycle and re-fix CO_2_ released by plastidial PDH during fatty acid biosynthesis (Schwender et al., 2004). The product of the RubisCO carboxylation reaction, 3-Phoshoglycerate (PGA) is then further processed to pyruvate via the plastidial glycolysis (**Figure 9**, No. 2). The advantage of such a pathway is that it results in a lower net loss of carbon as CO_2_. A prerequisite is the presence of the ribulose bisphosphate (RuBP) as CO_2_ acceptor in the stroma. Endosperm plastids contain enzymes of non-oxidative part of the OPPP (**Supplemental Table S4**), which together with the phosphoribulokinase (PRK, B9RZ33) can convert hexose phosphates to RuBP (**Figure 9**, No. 8).

Since sufficient oxygen is available in the endosperm after opening of the seed coat, including for the degradation of fatty acids by β-oxidation and the mitochondrial respiratory chain, the oxygenation reaction of RubisCO can occur (**Figure 9**, No. 1), leading to the formation of the toxic intermediate phosphoglycolate (PG). In photosynthetic tissues, the photorespiratory C_2_ cycle converts this molecule to PGA (**Figure 9**, No. 7), which requires the interaction of chloroplasts, peroxisomes, and mitochondria (Bauwe, 2023). The presence of peroxisomal glycolate oxidase in endosperms of castor beans germinated in the dark can be confirmed in our work (GOX, B9S0Y9), but is also documented in other studies (Cooper and Beevers, 1969; Schnarrenberger et al., 1971; Schmitz et al., 2020), implying that the oxidation of glycolate to glyoxylate take place in the peroxisomal lumen. Glyoxylate can be fed into the peroxisomal glyoxylate cycle to generate malate for sucrose biosynthesis. Feeding experiments with castor bean endosperm suggest a different fate for the glycolate (Cossins and Sinha, 1967). It is relatively rapidly converted to glyoxylate, glycine, serine, and CO_2_. This reaction sequence can be supported by our proteome data. Glyoxylate is transaminated to glycine by the peroxisomal glutamate/glyoxylate aminotransferase (GGAT, B9SPJ9) and then further processed in the mitochondria glycine decarboxylase complex (GDC, B9RRS7 and B9RXI7) and the serine hydroxymethyltransferase (SHMT, B9SMX7) to serine. The action of both mitochondrial enzymes is an essential source of one-carbon units, especially, for purine biosynthesis, while the glycine cleavage system releases CO_2_ and ammonium, which in turn can be re-assimilated by the endosperm plastid to compensate for losses. The additional enzymes of the photorespiratory pathway for the conversion of serine to PGA are absent from our endosperm proteome, but their activities of these missing proteins have been reported in the endosperms of castor beans germinated in the dark: Hydroxypyruvate reductase and serine/glyoxylate aminotransferase in peroxisomes (Schnarrenberger et al., 1971) as well as phosphoglycerate kinase in plastids (Benedict, 1973).

We found in the endosperm plastids numerous enzymes participating in the *de novo* synthesis of aromatic amino acids (phenylalanine, tryptophan, tyrosine), basic amino acids (lysine, arginine, histidine), branched-chain amino acids (leucine, isoleucine, and valine), aspartate, serine, methionine, threonine, glycine, and cysteine (**Supplemental Table S4**; **Figure 9**, No. 7). The plastid-localized biosynthesis of these amino acids is important throughout the germination period because most of the amino acids derived from storage proteins are already consumed at early stages of germination (Kermode et al., 1985; Gifford et al., 1986). Amino acids have a variety of tasks in the endosperm. Beside their major role as components of proteins, they serve as precursors for many primary and secondary metabolites, as alternative respiratory substrates for energy production (*e.g.*, BCAAs, Galili et al., 2014), and as nitrogen transport forms from endosperm to cotyledons (*e.g.*, glutamine and asparagine; Robinson and Beevers, 1981).

During storage protein mobilization, large quantities of ammonium (NH_4_^+^) are produced in the germinating castor bean seeds, which is also released by the mitochondrial glycine decarboxylase to a certain extend. As nitrogen in this form is toxic, it is reassimilated in the endosperm plastid via two conserved routes (Miflin and Habash, 2002): Ammonia and glutamate are converted into glutamine by the plastid glutamine synthetase (GS, B9RST2; **Figure 9**, No. 10) and then the amido group of the glutamine is transferred to a molecule of 2-oxoglutarate by the glutamate synthetase (GOGAT, B9RII5; **Figure 9**, No. 9), producing a net gain of one glutamate molecule. Additionally, ammonia can also be transformed into glutamine by NAD-dependent glutamate dehydrogenase (GDH, B9SLP5; **Figure 9**, No. 11). Glutamine and glutamate act as amino group donor for the synthesis of amino acids and nucleotides. The carbon skeleton for amino acid biosynthesis derived from intermediates of plastid metabolism, such as GS/GOGAT system (*e.g.*, glutamate, glutamine), non-oxidative part of the OPPP (*e.g.*, erythrose-4-phosphate, ribose-5-phosphate), and glycolysis (*e.g.*, PEP, 3-PGA). However, the carbon skeletons in germinating castor bean endosperm derived from storage oil are also used for synthesis of amino acids (Borek et al., 2011; Borek et al., 2013). Members of the RETICULATA protein family (B9SZJ5, B9RVJ6, and B9RIE6) known be involved in amino acid homeostasis have been found in our proteome for the cellular distribution of plastid-synthesized amino acids (Pérez-Pérez et al., 2013).

Plastids of the endosperm tissue contain the complete set of enzymes for the biosynthesis of adenosine monophosphate (AMP) during storage oil mobilization (**Supplemental Table S4**). At this stage of germination, the endosperm tissue contains the high levels of soluble nucleotides derived from plastid *de novo* synthesis, as the metabolic activity of the endosperm tissue is accompanied by increased gene expression (Kombrink and Beevers, 1983). At a later stage of seedling development, when the endosperm undergoes programmed cell death, the RNA and DNA are degraded to provide the embryo with their breakdown products (Schmid et al., 1999). AMP is exported from the plastids by the adenine nucleotide uniporter (BT1, B9RP65, unclassified 2x plastid, **Supplemental Table S3**) and hydrolyzed to adenosine in the cytosol for its transfer to the growing seedling (Flörchinger et al., 2006).

For their anabolic processes, plastids of the endosperm tissue rely on reduction equivalents and energy (Neuhaus and Emes, 2000). NADPH can be provided by the oxidative phase of the OPPP, converting glucose-6-phosphate to a pentose phosphate (Kruger and von Schaewen, 2003). Only the 6-phosphogluconate dehydrogenase (6PGDH, B9RVA7) as NADPH-producing step was identified, whereas NADP-dependent glucose-6-phosphate 1-dehydrogenase (G6PDH) and 6-Phosphogluconolactonase (6PGL) were missing in our plastid proteome (**Figure 9**, No. 6). Energy in the form of ATP is provided via the plastidial ATP/ADP (NTT, B9RUY6), importing cytosolic ATP generated via oxidative phosphorylation in exchange with ADP (Neuhaus et al., 1997). Surprisingly, we found some membrane-bound subunits of the plastidial ATP synthase (**Supplemental Table S4**). To date, there is no evidence that thylakoids are present in the stroma of plastids from endosperm, into which the ATPase can be inserted for ATP synthesis. Similarly, chromoplasts from tomato fruits possess a functional ATP synthase as an example for a non-photosynthetic plastid (Pateraki et al., 2013). This organelle is capable of synthesizing ATP via a respiratory electron transport chain using NADPH as an electron donor (Renato et al., 2015). However, components of such a respiratory pathway, like the thylakoid NAD(P) dehydrogenase and cytochrome b6f complex, which were present in chromoplasts from tomato fruits, were not found in our endosperm proteome (Barsan et al., 2010).

## 5 Conclusion

**Figure 10:**
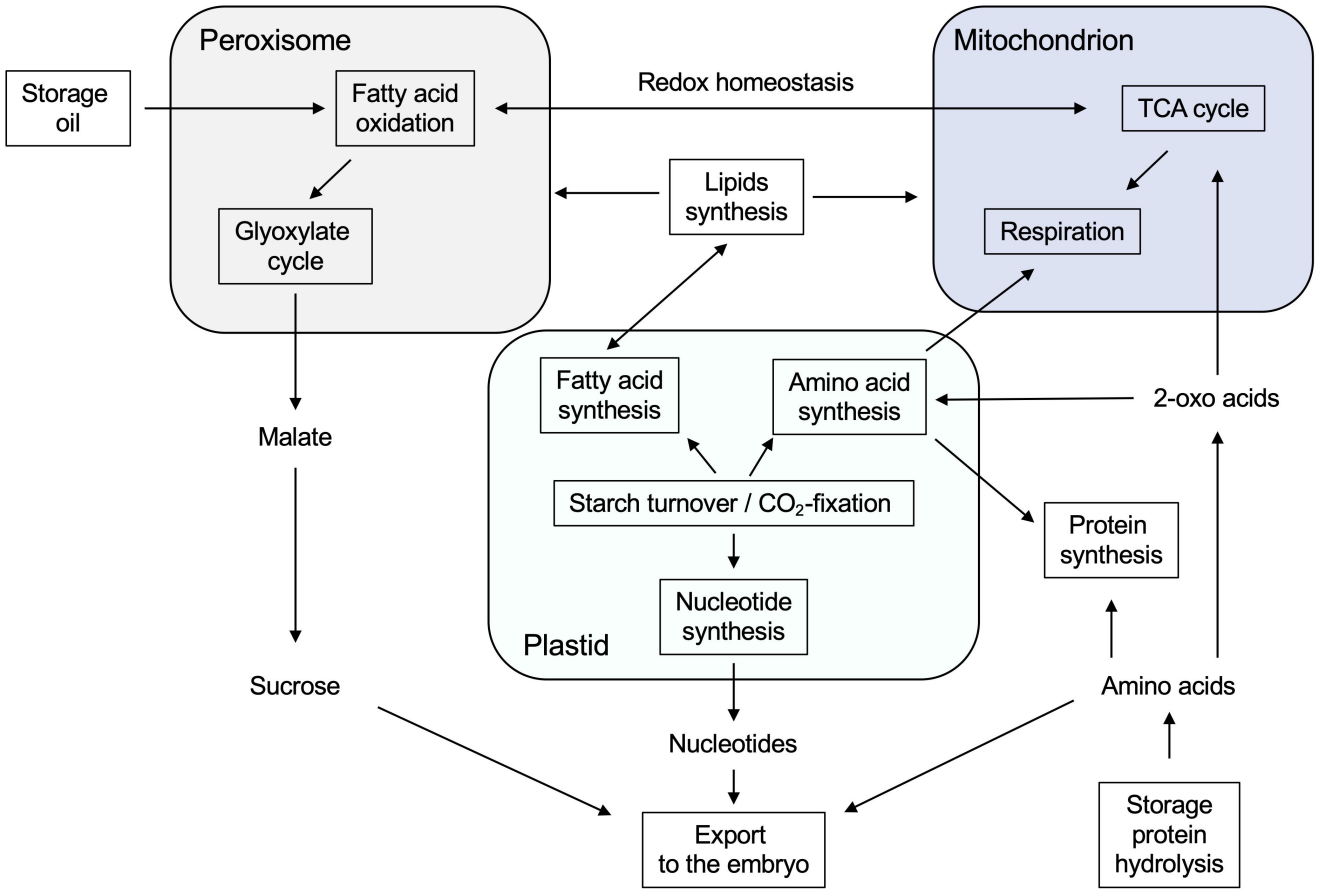
Metabolic network of peroxisomes, mitochondria, and plastids during storage reserve mobilization in germinating castor bean endosperm.

Overall, our endosperm proteome study revealed that mobilization of storage reserves, such as oil, proteins, and starch, is a multi-step process involving peroxisomes, mitochondria, and plastids in the endosperm tissue (**Figure 10**). Storage oil is converted into sucrose, which is exported to the embryo for its growth and development. The breakdown of seed-oil in the endosperm requires the cooperation of peroxisomes, mitochondria, and the cytosol. Storage proteins are hydrolyzed to amino acids in early stages of germination, which either remain in the endosperm for its own protein synthesis or are transferred to the embryo. At a later stage, however, plastids are the main source of amino acids, being able to synthesize amino acids *de novo*. Since most of the carbon skeletons from the stored oil goes into sucrose biosynthesis, mitochondria instead use amino acids as primary respiratory substrates.

Starch turnover in the endosperm plastids as well as the assimilation of CO_2_ by RubisCO serve as carbon sources for plastid-localized biosynthesis of amino acids, fatty acids, and nucleotides.

## Data availability statement

The mass spectrometry proteomics data have been deposited to the ProteomeXchange Consortium via the PRIDE partner repository with the dataset identifier PXD040932.

## Author contributions

TW conducted the experiments and analyzed the data. DB supported the data analysis. AS and KS performed the mass spectrometry-based proteome analysis. AM revised the manuscript. NL designed the experiments and wrote the manuscript. All authors contributed to the article and approved the submitted version.

## Supplementary material

Supplementary Table 1 List of proteins isolated from endosperm tissue of etiolated castor bean seedlings, which were identified and quantified in the density-gradient fractions containing the total proteins (T1 to T4) and membrane proteins (M1 to M4). Three biological replicates were subjected to proteome analysis. Only proteins contained at least two unique peptides and a minimum of three valid values in at least one fraction (total or membrane) were classified as “identified”. Values represent the absolute abundance of the proteins calculated by the sum of the peptide signal intensities.

Supplementary Table 2 List of castor bean proteins that have been defined as specific to peroxisomes, plastids, mitochondria, cytosol, vacuole, ER, Golgi, and nucleus based on (1) *in silico* prediction tools for their subcellular localization within the cell and (2) experimental data from GFP and/or MS assays of their corresponding Arabidopsis homologue using SUBA 5.0.

Supplementary Table 3 Deconvolution-based assignment of the MS-identified proteins from etiolated castor bean seedlings to their subcellular localization. Linear regression (LR), quadratic programming (QP), support vector regression (SVR), and non-negative matrix factorization (NMF) were applied to assign 2258 identified endosperm proteins to a specific cell compartment. The assignment of a subcellular compartment was made when at least three of the four deconvolution approaches resulted in a matching localization (consensus).

Supplementary Table 4 List of proteins found in peroxisomes, mitochondria, and plastids isolated from castor bean etiolated endosperm in this study. For functional annotation of proteins and their assignment to biological processes or metabolic pathways, gene ontology analysis was performed by Uniprot.

Supplementary Figure 1 Classification of the identified castor bean endosperm proteins from a soluble and membrane fraction enriched with peroxisomes **(A)**, mitochondria **(B)**, and plastids **(C)**. The classification is based on UniProt database. Numbers in percent refer to the number of proteins that have been assigned to a certain metabolic pathway relative to the total proteins of all metabolic pathways of the respective organelle.
