# Supplementary figures and images for: Mapping the castor bean endosperm proteome revealed a metabolic interaction between plastid, mitochondria, and peroxisomes to optimize seedling growth
Source: Front Plant Sci. 2023 Oct 6;14:1182105. doi: 10.3389/fpls.2023.1182105 (PMC10588648; doi:10.3389/fpls.2023.1182105)

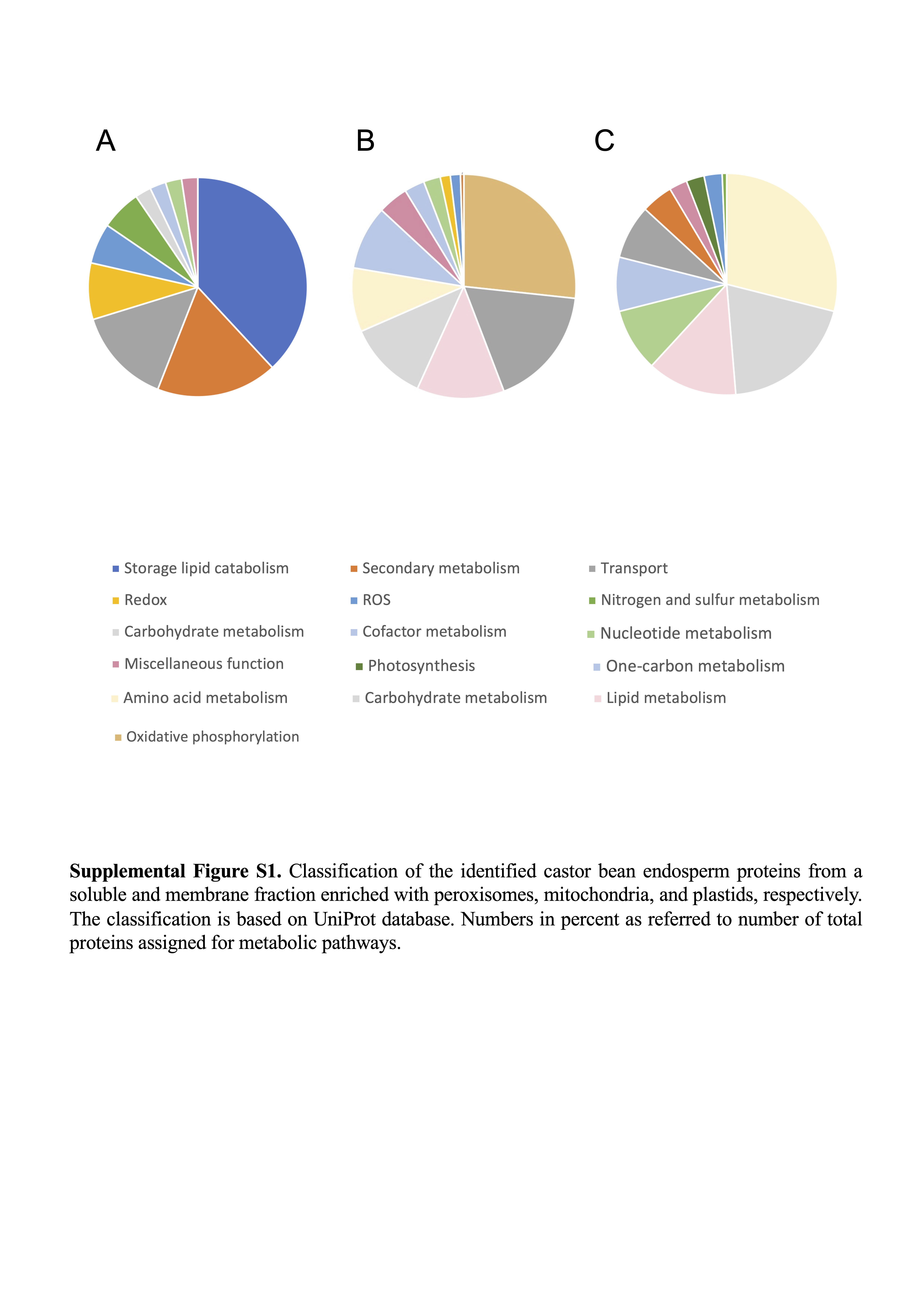

Supplement: Supplementary Figure 1 — Classification of the identified castor bean endosperm proteins from a soluble and membrane fraction enriched with peroxisomes (A), mitochondria (B), and plastids (C). The classification is based on UniProt database. Numbers in percent refer to the number of proteins that have been assigned to a certain metabolic pathway relative to the total proteins of all metabolic pathways of the respective organelle. [file Image_1.jpg]
